# Cancer Stemness: p53 at the Wheel

**DOI:** 10.3389/fonc.2020.604124

**Published:** 2021-01-11

**Authors:** Dishari Ghatak, Damayanti Das Ghosh, Susanta Roychoudhury

**Affiliations:** ^1^ Cancer Biology and Inflammatory Disorder Division, CSIR-Indian Institute of Chemical Biology, Kolkata, India; ^2^ Division of Research, Saroj Gupta Cancer Centre and Research Institute, Kolkata, India

**Keywords:** GOF mutant p53, cancer stemness, differentiation, epithelial to mesenchymal transition, chemoresistance, miRNAs, therapeutic targeting

## Abstract

The tumor suppressor p53 maintains an equilibrium between self-renewal and differentiation to sustain a limited repertoire of stem cells for proper development and maintenance of tissue homeostasis. Inactivation of p53 disrupts this balance and promotes pluripotency and somatic cell reprogramming. A few reports in recent years have indicated that prevalent *TP53* oncogenic *gain-of-function* (GOF) mutations further boosts the stemness properties of cancer cells. In this review, we discuss the role of wild type p53 in regulating pluripotency of normal stem cells and various mechanisms that control the balance between self-renewal and differentiation in embryonic and adult stem cells. We also highlight how inactivating and GOF mutations in p53 stimulate stemness in cancer cells. Further, we have explored the various mechanisms of mutant p53-driven cancer stemness, particularly emphasizing on the non-coding RNA mediated epigenetic regulation. We have also analyzed the association of cancer stemness with other crucial *gain-of-function* properties of mutant p53 such as epithelial to mesenchymal transition phenotypes and chemoresistance to understand how activation of one affects the other. Given the critical role of cancer stem-like cells in tumor maintenance, cancer progression, and therapy resistance of mutant p53 tumors, targeting them might improve therapeutic efficacy in human cancers with *TP53* mutations.

## Introduction

The tumor suppressor p53 has been described as the “guardian of the genome” for its pivotal role in protecting the cells from neoplastic transformation. Apart from its classical function in cell-cycle arrest, DNA-repair, apoptosis, and senescence, it also supervises processes such as cellular plasticity, self-renewal, and differentiation (1, 2). *TP53* maintains homeostasis between self-renewal and differentiation depending on the cellular and developmental state and prevents the dedifferentiation and reprogramming of somatic cells to stem cells (2). *TP53* is frequently altered in human tumors. The majority of alterations are somatic missense mutations that occur in the DNA binding domain between amino acids 125 to 300 (3). The DNA-binding domain mutants are categorized into “contact” (R248, R273) mutants, where amino acid residues involved in making direct contact with the DNA and “conformational” mutants (R175H, G245, R249, and R282) that disrupt the p53 protein structure at a local or global scale (4, 5). These mutants not only lose the canonical tumor-suppressive functions of their wild-type counterpart but also empower cancer cells by imparting gain-of-function (GOF) properties that favor cancer cell survival and promote tumor progression (6–9).

The GOF mutant p53 proteins regulate several cellular genes and non-coding RNAs primarily as a transcription factor and confer oncogenic properties such as sustained proliferation, increased chemoresistance, invasion and metastasis, angiogenesis, deregulated cellular metabolism, genomic instability, resistance to cell death, evading immune destruction, and replicative immortality (10). In recent years, a novel function of mutant p53 in promoting dedifferentiation of somatic cells to cancer stem cells (CSCs) has gathered considerable attention. The notion that GOF mutant p53 play a major role in CSC formation was derived from the undifferentiated and chemoresistant nature of the mutant p53 tumors (11). This was further supported by the common gene signature and similar transcription factor shared among embryonic stem cells (ESCs) and undifferentiated tumors of breast and brain (12). The poor prognosis of cancer patients with p53 mutations also strengthened this belief (13). However, a few direct evidence supporting the role of mutant p53 in driving CSC phenotype came along only in the recent years (14, 15). In this review we discuss various mechanisms driving alteration of cellular plasticity upon p53 mutation and efforts to delineate novel ways to specifically target the aggressive CSCs residing in mutant p53 tumors or to obstruct mutant p53 driven conversion of somatic cells to CSCs.

## Stem Cells and Cancer Stem Cells

Stem cells are a rare population of cells that can perpetuate themselves through self-renewal and can give rise to mature cells of a tissue by differentiation (16). While embryonic stem cells (ESCs) are pluripotent and have the ability to differentiate into three embryonic lineages, ectoderm, mesoderm, and endoderm, adult stem cells (ASCs) being multipotent in nature can differentiate into cells of a particular lineage. For example, hematopoietic stem cells (HSCs) can generate cells of the hematolymphoid system only (16). Stem cells in tissues reside in a specific location and are responsible for homeostasis and maintenance of tissue integrity and repair of damaged tissue.

Cancer stem cells (CSCs) are a subset of tumor cells that can self-renew and differentiate to generate the heterogenous cell population in a tumor (16). CSCs and normal stem cells share the ability of persistent proliferation that maintain the CSC/stem cell pool and also generate differentiated cells that form the bulk of tumor/tissue. The heterogeneity in solid tumors has been explained by two main models. The “stochastic” or “clonal evolution” model suggests that every cancer cell present in a tumor possess the same potential to proliferate and generate a new tumor (17). On the contrary, the “hierarchical” model postulates a hierarchical organization of cells in a tumor, with a subpopulation of cells accountable for maintenance of heterogeneity in primary tumor and generating new tumors similar to the original one (16, 18, 19). This population of tumor initiating cells has been termed as cancer stem cells for their “stem-like” ability of self-renewal and differentiation.

Although, the “hierarchical” model has been widely adopted but some evidences suggest that this template is not applicable for all adult stem cell/cancer stem cell prototypes. The hierarchical model suggests that stem cells/CSCs are rare and quiescent, however, the adult stem cells residing in epidermis or intestinal crypts are abundant in their niches and can actively divide throughout their lives (20). According to the “hierarchical model” stem cells/CSCs undergo asymmetric division to form one stem cell and one daughter cell (21). However, some adult stem cells can divide to generate zero, one, or two new stem cells which compete to occupy the niche by a process called neural competition (22, 23). Moreover, these adult stem cell hierarchies are extremely plastic, implying that the daughter cells and fully differentiated cells can revert to form stem cells and occupy the niche. For example, the differentiated hepatocytes can re-enter the cell cycle and can replace lost tissue upon hemi-hepatectomy (24).

CSCs and non-CSCs undergo transitions between stem and differentiated state upon exposure to therapeutic insults or certain stimuli within the microenvironment (25–27). For example, upon radiation treatment CSCs are enriched *in vivo* which suggests that radiation induces phenotypic transition of non-CSCs to CSCs. Similarly, cisplatin treatment triggers ovarian cancer non-CSCs to acquire self-renewal property (27). Furthermore, differentiated colorectal cancer cells were found to give rise to CSCs upon NF-kB activation, APC depletion, and upon chemically induced inflammation (28, 29). The dynamic nature of the CSCs and non-CSCs were further exemplified by the study in which cell population isolated based on stem cell, basal or luminal like phenotype from a breast cancer cell line could undergo phenotypic transitions *in vitro* and generate cells of the other two types (30). Interestingly, all the subcultures grown from all the three subpopulations converged over time to the same proportion of cell types of the original breast cancer cell line indicating that the inter-conversions were stochastic and independent of the phenotype of the cell of origin. However, the phenotypes were functionally significant as only the stem-like cells formed tumors upon xenotransplantation. Cell ablation experiments have recently been used to investigate CSC plasticity in human cancer xenografts (31, 32). Using CRISPR-Cas9 approach, inducible caspase 9 (iCasp9) was inserted in the *LGR5* locus of human colorectal cancer organoids, which is a common CSC marker for colorectal cancer (31). The induction of apoptosis in xenografts produced by these organoids resulted in shrinkage of tumor. However, upon removal of the inducer, the mitotically arrested, differentiated tumor cells restored the Lgr5+ CSC population and proliferated to regenerate the tumor. This further establishes the plasticity of CSC and non-CSC population in tumors. However, in certain cancer types the hierarchical organization is proposed to be unidirectional and largely irreversible. The ablation of CSC pool in glioblastoma xenograft halted tumor growth without apparent regeneration of the CSC pool from the other non-CSC glioblastoma cells (33). Although CSCs share the core traits of self-renewal and differentiation with normal stem cells, the phenotypes of the CSCs are more complex, varying from one tumor to another and are influenced by the abnormalities occurring during neoplastic transformation.

## Wild Type p53 Controls Cellular Plasticity

Apart from the acclaimed role of p53 as the “guardian of the genome” in somatic differentiated cells, a profound function of it has also been established in stem cells. Recent studies combined with the basic information obtained in last 25 years provide an understanding of how wild-type p53 regulate the quantity and quality of stem cells to ensure normal development and a cancer-free life. In this section we address the role of p53 in regulating embryonic stem cell and adult stem cell self-renewal and differentiation, in preventing CSC formation and in generation of induced pluripotent stem cells.

### p53 Controls the Balance Between Self-Renewal and Differentiation in Embryonic Stem Cells

**Figure 1:**
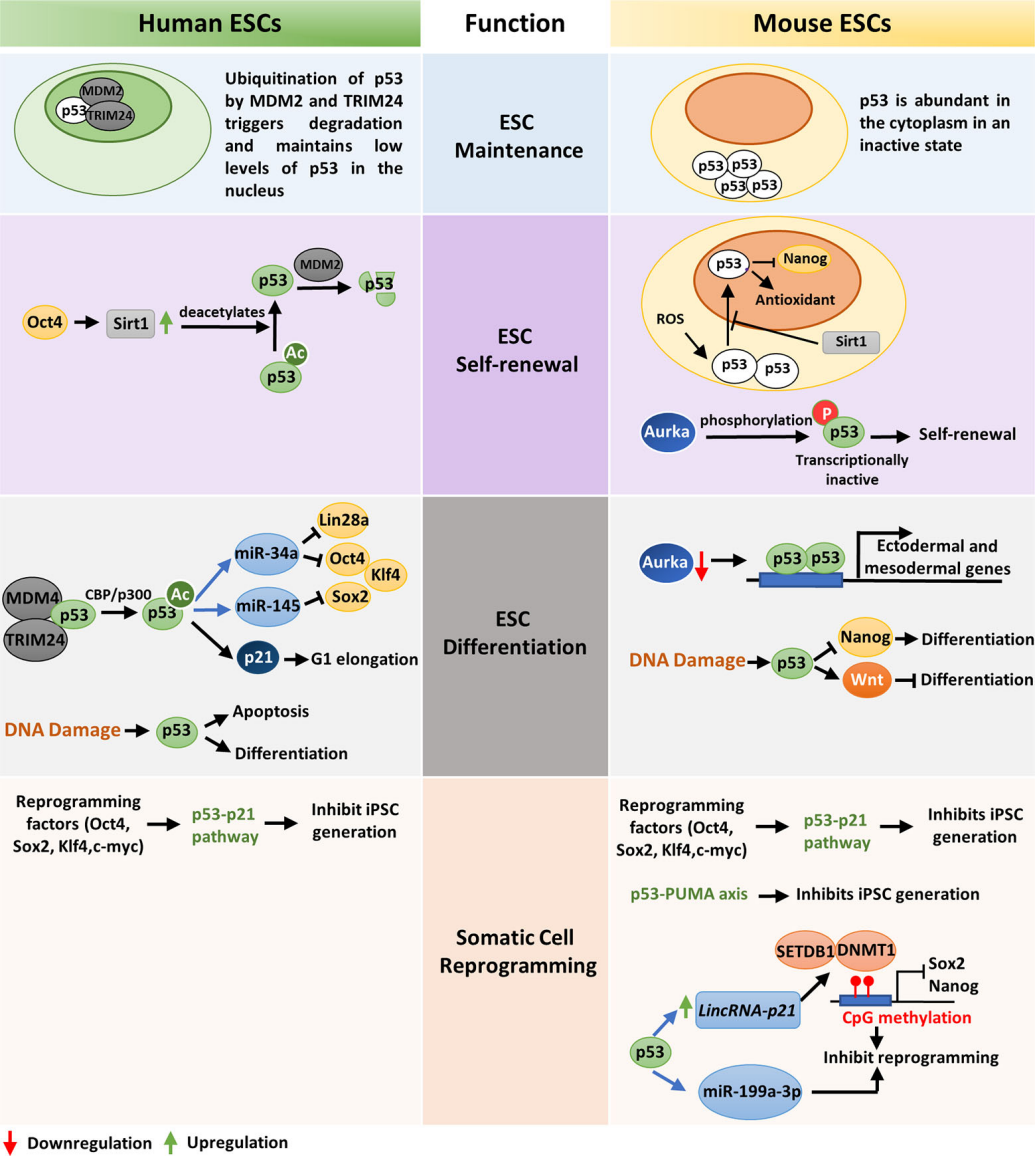
A comparative view of wild-type p53 function in ESC maintenance, differentiation, and somatic-cell reprogramming of human and mouse: *ESC maintenance*: p53 is maintained in an inactive state in both human and mouse ESCs. In hESCs, deacetylated inactive p53 is present in low levels in the nucleus while in mESCs the inactive p53 protein is abundantly present in the cytoplasm. *ESC self-renewal*: To ensure ESC self-renewal, p53 is either prevented from entering the nucleus or maintained in an inactive state. In hESCs, Oct4 increases Sirt1 expression which in turn deacetylates p53 and promote its degradation by MDM2. This maintains a low level of p53 in the cell which is crucial to maintain stemness. Endogenous ROS induced p53 nuclear translocation in mESCs is blocked by Sirt1. This prevents p53 mediated suppression of Nanog and stem-cell phenotype is maintained. Phosphorylation and subsequent inactivation of p53 by Aurka also promotes pluripotency of mESCs. *ESC differentiation*: In hESCs, CBP/p300 mediated acetylation of p53 leads to its activation and subsequent transcription of p21, miR-34a and miR-145 which facilitates differentiation. DNA damage in hESCs also leads to differentiation or apoptosis. When Aurka levels are low in mESCs, p53 transcribes ectodermal and mesodermal genes leading to differentiation. Also, upon DNA damage, p53 primarily promotes differentiation by suppression of Nanog. However, occasionally p53 may also induce anti-differentiation pathway by activating Wnt. *Somatic-cell reprogramming*: Reprogramming of somatic cells to induced pluripotent stem cells (iPSCs) is primarily inhibited by the p53-p21 pathway in both human and mouse. Additionally, p53 may also induce lincRNAp21 or miR-199a-3p to inhibit reprogramming. The p53-PUMA axis has also been found to suppress reprogramming of mouse embryonic fibroblasts (MEFs).

The tumor suppressor p53 plays a significant role in ensuring genomic integrity of embryonic stem cells and controls their proliferation, differentiation, and apoptosis. In human embryonic stem cells (hESC), p53 is present in low levels due to the negative regulation by E3 ubiquitin ligases HDM2 and TRIM24 (**Figure 1**). Acetylation of p53 at K373 by CBP/p300 leads to dissociation of HDM2 and TRIM24 and subsequent activation of p53 which in turn transcriptionally activates p21, miR-34a, and miR-145 (**Figure 1**). Induction of p21 elongates G1 phase facilitating differentiation while, miR-34a and miR-145 counteracts pluripotency by targeting Lin28a, Oct4, Klf4, and Sox2 (**Figure 1**) (34). Similarly, p53 activation by nutlin leads to transcriptional activation of p21 that cause cell cycle arrest and induces differentiation in human ESCs (35). As activation of p53 leads to differentiation of ESCs, p53 is maintained in an inactive state during self-renewal of human ESCs by Oct4 induced Sirt1 mediated deacetylation (**Figure 1**) (36).

Unlike human ESCs, mouse ESCs display high levels of p53 protein localized in the cytoplasm, which declines during organogenesis and is barely detected in terminally differentiated tissues (**Figure 1**) (37). When mESCs are exposed to reactive oxygen species (ROS), Sirt1 facilitates translocation of p53 to the mitochondria instead of nucleus and induces mitochondrial-dependent apoptosis. This blocks p53 mediated suppression of Nanog transcription and maintains ESC pluripotency (**Figure 1**) (38). Lee et al. showed that in mouse embryonic stem cells (mESCs), Aurka-mediated phosphorylation of p53 suppress p53 activity and mediates mESC pluripotency (**Figure 1**). However, when Aurka levels are low, p53 transcriptionally activates ectodermal and mesodermal genes leading to differentiation (**Figure 1**) (39). Sabapathy et al. found that undifferentiated embryonic stem cells derived from murine embryonic stem cell lines express high levels of p53 in wild type conformation. *In vitro* differentiation of these cells resulted in decrease of p53 protein and triggered a shift in its conformation to mutant form (40).

DNA damage in embryonic stem cells leads to p53 activation and subsequent differentiation (41). In hESCs DNA lesions trigger p53-dependent apoptosis and differentiation (42). Although the role of p53 in DNA damage repair in ESCs is debatable, p53 deletion has been found to increase ESC survival upon DNA damage (43, 44). DNA damage in mESCs leads to activation of p53 by phosphorylation at Ser 315 residue, which then binds to the promoter of ESC self-renewal gene Nanog and suppresses its transcription (**Figure 1**) (45). This induces differentiation of mESCs and maintains their genomic stability. Apart from DNA damage, oncogenic stress signals and stimuli such as retinoic acid also induce differentiation of mESCs (46). Interestingly, p53 has also been found to induce anti-differentiation programs in mouse ESCs in response to UV radiation mediated DNA damage by directly regulating the Wnt pathway (**Figure 1**) (47). This suggests that p53 is a crucial regulator of both pro-differentiation and anti-differentiation programs and maintains homeostasis between self-renewal and differentiation depending on the developmental state (47). The role of p53 as a pluripotency switch was elaborately explored by Ungewitter et al. (48). They found that partial expression of p53 isoform Δ40p53 led to loss of pluripotency in mouse ESCs and triggers differentiation in somatic cells. However, increased expression of Δ40p63 isoform helped in stem cell maintenance mediated by Nanog and IGF-1 receptor and other p53 family members, p63 and p73 (41). Although p53 knockout mice grow normally, they develop tumors in their adult life which suggests that p53 is involved in assuring the genetic fidelity in embryonic stage (49). The critical role of p53 in embryonic development is further supported by the developmental defects, low fertility, and spermatogenesis defects exhibited by p53 null mice (50, 51).

### p53 Acts as a Barrier to Somatic Cell Reprogramming

Somatic cells can be reprogrammed to induced pluripotent stem cells (iPSCs) by overexpression of transcription factors such as *OCT4*, *SOX2*, *KLF4*, and *c-MYC* (52, 53). These factors, also known as Yamanaka factors, are highly expressed in embryonic stem cells and regulate the developmental signaling required for ES cell pluripotency. However, the efficiency of somatic cell reprogramming is considerably low, and very few cells are reprogrammed to iPSCs (54). A recent study by Zhao et al. demonstrated that siRNA mediated knockdown of p53 in human adult fibroblasts enhance the efficiency of iPS cell generation up to 100-fold even in the absence of *c-MYC* overexpression (55). Also, reduction of p53 signaling by knocking down its target gene p21, or antagonizing apoptosis induced due to reprogramming, increases efficiency of transformation (56). Functional analysis of common set of genes expressed in mouse and human fibroblasts revealed p53-p21 pathway as the roadblock to iPS cell generation (**Figure 1**) (57). Indeed, the expression of reprogramming factors activates p53 pathway which eliminates cells with DNA damage, DNA repair deficiencies and those with shortened telomeres by the activation of DNA damage response or p53-dependent apoptosis (58). However, when p53 is abrogated, somatic cells carrying persistent DNA damage or chromosomal aberrations are efficiently reprogrammed to iPS cells. This indicate that reprogrammed cells are tolerant to different types of DNA damage and p53 act as a barrier in generation of human and mouse iPS cells from suboptimal parental cells. The pro-apoptotic protein PUMA has also been found to be an independent facilitator of p53 mediated suppression of induced pluripotent stem cell generation (**Figure 1**) (59). p53 may also impede reprogramming by inducing lincRNAp21 which associates with H3K9 methyltransferase SETDB1 and DNA methyltransferase DNMT1 and maintains CpG methylation at Sox2 and Nanog promoters (60). Moreover, p53 may upregulate miR-199a-3p which in turn impose G1 arrest, to decrease reprogramming efficiency (**Figure 1**) (61). Although permanent suppression of p53 during iPS cell generation may have deleterious effects on the genomic stability of the reprogrammed cells, transient knockdown of p53 may be useful in efficiently producing integration-free iPS cells for future medical use (62).

### p53 Promotes Differentiation in Adult Stem Cells

**Figure 2:**
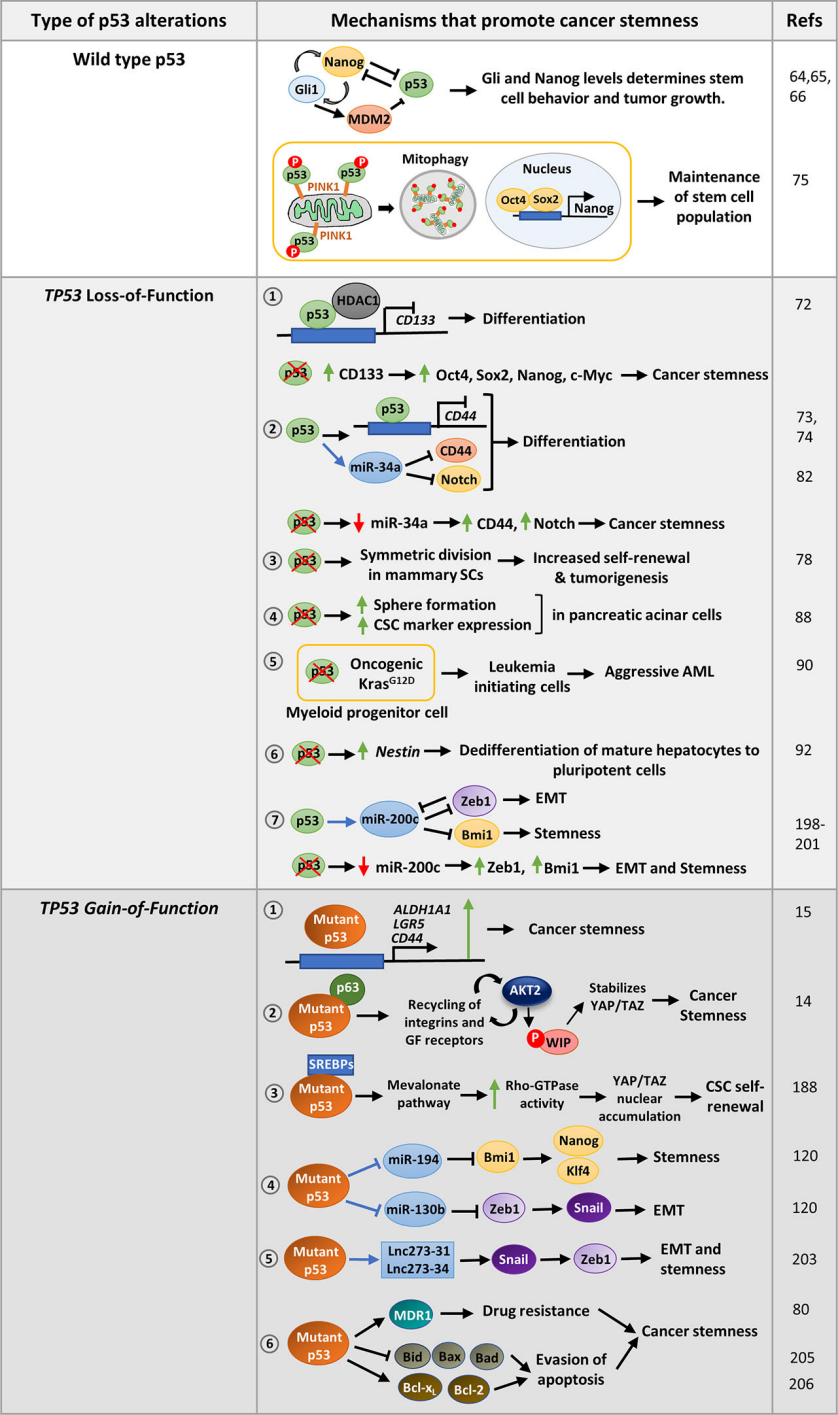
Mechanisms that promote stemness in cancer cells harboring wild-type p53, p53 with loss-of-function mutations or gain-of-function missense mutations: Wild-type p53 modulates the Nanog -Gli positive feedback loop in neural stem cells to control pluripotency. On the contrary, Nanog suppresses p53 activity while Gli activated by Nanog inhibits p53 by activating Mdm2 to promote pluripotency. In hepatic cancer, the stem cell population is maintained by removing mitochondria-associated p53 through mitophagy. *TP53* LOF mutations promote various mechanisms that confer stemness phenotype to cancer cells. 1. p53 loss upregulates CD133 which subsequently promotes CSC marker expression and confers stemness. 2. p53 suppresses the cell-surface marker CD44 either by binding to its promoter or by upregulating miR-34a. p53 loss results in increased expression of CD44 and Notch leading to cancer stemness. 3. Loss of p53 also promotes symmetric division of mammary SCs thereby promoting tumorigenesis. 4. Homozygous deletion of p53 in pancreatic acinar cells promotes sphere formation, CSC marker expression as compared to cells with wild type p53. 5. p53 inactivation strongly cooperates with oncogenic Kras mutation in myeloid progenitor cells to induce aggressive AML. 6. p53 loss may also derepress SC marker Nestin to promote differentiation in mature hepatocytes. 7. p53 induces epithelial differentiation by activation of miR-200c. Loss of p53, leads to decreased miR-200c levels and increased expression of its target genes leading to EMT and stemness. *TP53* GOF mutations promote cancer stemness by regulating several pathways. 1. Mutant p53 can directly activate CSC markers such as ALDHA1, CD44, and LGR5 to promote stemness. 2. It may regulate Wasp-interacting protein (WIP) that regulates YAP/TAZ stability. 3. Mutant p53 can also promote self-renewal of breast cancer cells by inducing nuclear localization of YAP/TAZ by activating mevalonate pathway. 4. Mutant p53 transcriptionally represses miR-130b and miR- 194, the negative regulators of Zeb1 and Bmi1 respectively, to promote EMT and stemness 5. p53-R273H upregulates lncRNAs, lnc273-31, and lnc273-34 implicated in EMT and CSC maintenance in colorectal cancer cells. 6. GOF mutant p53 promotes typical CSC features of enhanced drug-resistance and prolonged survival by upregulating multidrug resistance gene MDR1, anti-apoptotic genes Bcl-2 and Bcl-xL, and inhibiting pro-apoptotic genes Bax, Bid, and Bad.

*TP53* is also a critical regulator of adult stem cell differentiation. Zheng et al. reported that downregulation of Myc by the cooperative actions of p53 and PTEN is crucial for differentiation of murine neural stem cells (NSCs) (63). p53 was found to control proliferation of NSC through inhibition of Gli activity and nuclear localization, the effector of hedgehog signaling pathway (**Figure 2**) (64). Gli in turn repress p53 by activation of Mdm2, forming a homeostatic inhibitory loop (64). The hedgehog signaling pathway can also drive self-renewal through activation of Nanog which is otherwise suppressed by p53 (**Figure 2**) (65, 66). Altogether, the Nanog-Gli-p53 axis determines NSC self-renewal and differentiation. In p53-deficient mouse astrocytes Nanog is uninhibited and promotes dedifferentiation to produce cancer stem-like cells (67). p53 deficiency also elevate the rate of neurosphere formation from the olfactory bulb cells of mouse embryo indicating that self-renewal is enhanced by loss of p53 (68). p53 also play a crucial role in regulating self-renewal and differentiation of mesenchymal stem cells (MSCs) (69). MSCs derived from p53KO mice show augmented proliferation, increased differentiation rate, and a predisposition to transformation (70). Although primary mouse bone marrow stromal cells (mBMSCs) derived from wild-type p53 or p53 knockout mice have differentiating capacity into osteogenic, adipogenic, and chondrogenic lineages, enhanced osteogenic differentiation has been found only in the absence of p53 (71). This is due to increased levels of Runx2 in p53 knockout mice, which remains suppressed by the elevated expression of miR-34 family in wild type p53 cells (71). Hence p53-deficient mBMSCs are more closely related to human osteosarcoma (71).

Wild type p53 has also been found to compromise CSC properties by directly repressing CSC markers or indirectly by inducing certain miRNAs. For example, p53 repress CD133 by directly binding to its promoter and recruiting HDAC1 (**Figure 2**). Depletion of CD133 suppresses core stemness factors Oct4, Nanog, Sox2, and c-Myc and promotes differentiation (72). Likewise, p53 suppress tumor formation by inhibiting the expression of the CSC marker CD44 by binding to a noncanonical p53-binding site on its promoter (**Figure 2**) (73). Further, induction of miR-34a by p53 functionally targets the CSC marker CD44, thereby inhibiting prostate cancer regeneration and metastasis (**Figure 2**) (74). To facilitate pluripotency, cancer stem cells keep wild type p53 levels in control. For instance, the hepatic cancer stem cell population is maintained by removal of mitochondria by autophagy. This eliminates mitochondria-associated p53 which would otherwise be activated by PINK1 to mediate suppression of Nanog (**Figure 2**) (75). Interestingly, Flesken-Nikitin et al. found that alteration of p53 status of cancer-prone SCs residing in ovarian-surface epithelium enhanced their transformation potential (76). To prevent oncogenic transformation, p53 activity is maintained by certain proteins like NUMB, a cell-fate determinant and tumor suppressor. Apart from promoting asymmetric cell division, NUMB associates with p53 and MDM2 in a tricomplex preventing ubiquitination and degradation of p53 (77). Hence, loss of NUMB in breast cancer cells leads to decreased p53 levels and increased activity of NOTCH receptor which confers increased chemoresistance (77). In a similar study, loss of p53 in mammary SCs was found to promote symmetric cell-divisions leading to increased self-renewal property and subsequently contribute to tumorigenesis (**Figure 2**) (78). Further, the human p53 isoform Δ133p53β lacking the transactivation domain was observed to promote CSC features in breast cancer cell lines by expression of Sox2, Oct3/4, and Nanog in a Δ133p53β dependent manner (79).

Further, p53 sensitizes cells to drug induced apoptosis by downregulating the multidrug resistance gene, *MDR1* (80, 81). Additionally, p53 upregulates miR-34a that represses Notch (**Figure 2**) and anti-apoptotic Bcl2 thereby promoting differentiation and apoptosis (82). Therefore, it can be concluded that wild type p53 functions to maintain a balance between self-renewal and differentiation to maintain tissue integrity, which is lost upon p53 mutation.

## p53 Mutation Imparts Stem-Like Properties to Cancer Cells

Any mutation of p53 (deletion and GOF missense) have the loss of wild-type function as the first consequence. The loss of tumor suppressive functions of p53 triggers multipotent/unipotent adult cells to dedifferentiate and acquire pluripotency which results in disturbances in tissue hierarchy. With the advent of reprogramming era, it was further highlighted that p53 loss promote dedifferentiation and reprogramming under favorable conditions. p53 inactivating mutations in tumors results in increased expression of CSC markers and sphere forming ability. Certain p53 missense mutants further promote these phenotypes aggravating the malignant condition.

### p53 Inactivation Leads to Cancer Stemness

Although majority of tumors harbor p53 *loss-of-function* mutation (missense and truncation mutations) or functional inactivation of p53 pathway, it is more prominently correlated with dedifferentiated sarcomas and carcinomas (83). For instance in breast cancer, p53 mutation is frequently correlated with high-grade tumor types including poorly differentiated basal-like tumors (84–87). Pinho et al. revealed that pancreatic acinar cells with homozygous deletion of p53 show stemness features such as enhanced sphere formation, increased expression of CSC markers (Ptf1a,Pdx1, Cpa1, c-Myc, Sox9, and Hnf1b) and stem cell regulators like Bmi1 and Klf4 as compared to cells with wild type p53 (**Figure 2**) (88). In accordance, a later study demonstrated that p53-miR-200 axis negatively regulates Sox2, and counteracts NFATC1-Sox2 mediated dedifferentiation of pancreatic adenocarcinoma cells (89). Association of p53 inactivation and loss of differentiation characteristics has also been reported in AML and lung cancer (**Figure 2**) (90, 91). Furthermore, p53 loss was found to trigger dedifferentiation of mature hepatocytes to pluripotent cells by the activation of SC marker *Nestin*, which remains suppressed in wild-type p53 bearing cells (**Figure 2**) (92). Mammary stem cells with p53−/− and p53+/− formed larger and more number of mammospheres compared to p53+/+ cells (93). Moreover, tissue-specific adult stem cells of mouse mammary epithelium, which are not pluripotent but maintain tissue homeostasis, become tumorigenic in presence of p53 deletion (78). An interesting study by Mizuno et al. propounded that breast and lung tumors with functionally compromised wild-type p53 have gene-expression pattern like ESCs (84). They also observed that breast tumors with very low ARF levels correlated with high scores for ESC signature. As ARF inhibits MDM2, low level of ARF results in high MDM2 activity and low levels of p53 which induce the SC phenotype. ARF has been found to be repressed by the polycomb complex protein Bmi1 that maintains stem cell self-renewal by maintaining low p53 protein level (94). Besides, loss of downstream effector p21 also enhances tumorigenesis in p53 deleted stem cells (95). In light of these observations one can speculate that p53 loss promote expression of a set of genes that cause reversion of the cells from terminally differentiated state to a more stem-like state that enhance tumor growth. Even when p53 is functional, deregulation of genes modulating p53 pathway can also trigger a similar phenotype.

### p53 Gain-of-Function Mutation Promotes Cancer Stemness

The most frequently occurring mutations in p53 are missense point mutations that cluster in the DNA binding domain region. There are six amino acid residues, termed as “hotspots,” which are commonly altered by such mutations. These mutations not only result in loss of tumor suppressive functions of p53 but also promote several oncogenic phenotypes. Hence, they are known as “*gain-of-function*” (GOF) mutations. Although the GOF mutant protein lack DNA-binding ability, they can piggyback on other transcription factors to regulate expression of a large number of genes and non-coding RNAs. In this section we will discuss the different oncogenic properties conferred by GOF mutant p53 and its role in regulating stemness of cancer cells.

#### Oncogenic Properties of GOF Mutant p53

The GOF mutant p53 proteins can sense the extrinsic and intrinsic stress conditions of transformed cells and synchronize adaptive responses that support tumor growth and sustenance (96). These proteins help cancer cells to cope with stress generated during tumorigenesis, such as hyperproliferation induced DNA damage, oxidative and proteotoxic stress, physical constraints, nutrient fluctuations, stromal cues, and anti-tumor immune response by promoting oncogenic *gain-of-function* phenotypes (96).

**Figure 3:**
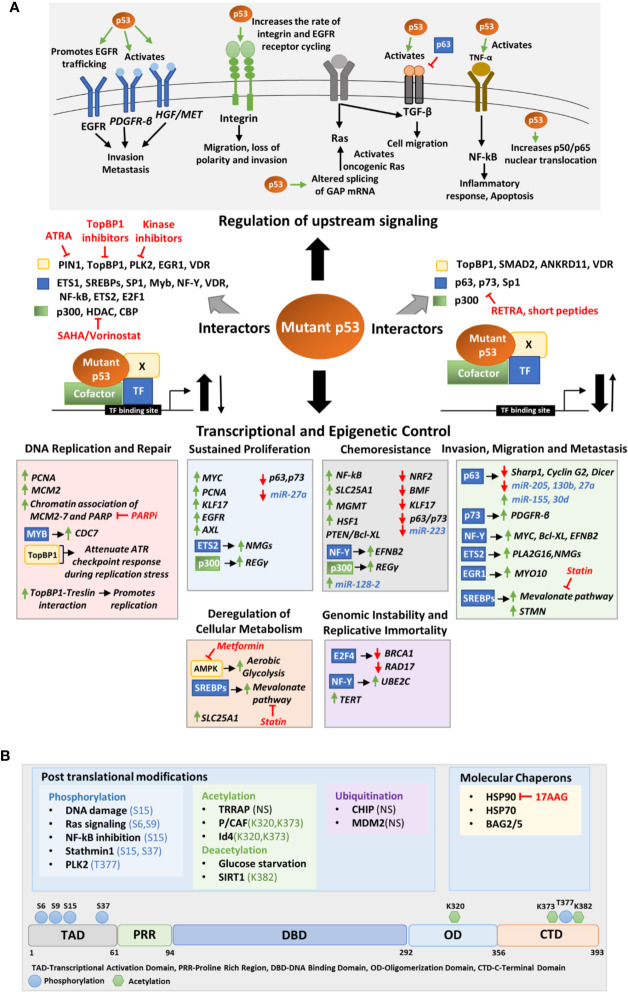
**(A)** Molecular mechanisms of mutant p53 mediated deregulation. The upper panel depicts the upstream signaling pathways deregulated by mutant p53 to promote oncogenesis. The middle panel portrays the different transcription factors, cofactors, and other proteins to which mutant p53 may interact to either enhance or inhibit their binding to the target gene promoter. The lower panel shows the transcriptional and epigenetic targets of mutant p53 classified according to the phenotype they alter. **(B)** Upstream signals that regulate mutant p53. The upper panel shows the various post translational modifications and chaperons that regulate mutant p53 stability. The modified residues if known, have been mentioned. In others it is not-specified (NS). The lower panel shows the residues in the mutant p53 protein where post-translational modifications occur. Drugs that target interacting proteins of mutant p53, downstream pathways and upstream regulators have been indicated in red in both panels **(A, B)**.

One of the distinctive stress responses of mutant p53 bearing cells is their ability to resist cell death as well as chemotherapeutic drugs insults (97, 98). This gain-of-function property of mutant p53 was revealed in 1995 when Lotem and Sachs observed that mutant p53 expression could inhibit c-Myc induced apoptosis in leukemic cells (99). Various proteins and signaling pathways are implicated in mutant p53 mediated resistance to chemotherapeutic drugs. For instance, mutant p53 driven activation of NF-kB (**Figure 3A**) or increased expression of MGMT or SLC25A1 (**Figure 3A**) confer increased resistance to etoposide, temozolomide, and cisplatin, respectively (100–102). Further, mutant p53 can interact with PELP1 to promote resistance to platinum-based drugs in triple negative breast cancer (103). A recent study by Alam et al. reveals GOF mutant p53 upregulates EFNB2 and activates ephrin B2 reverse signaling to impart enhanced chemoresistance to colorectal cancer cells (**Figure 3A**) (104). Mutant p53 also protects the cancer cells from oxidative and proteotoxic stress. For instance, it suppresses NRF2 which regulates the expression of antioxidant proteins (**Figure 3A**) (105). Mutant p53 also promotes the function of HSP1 by direct (binding) or by indirect (EGFR/ErbB2 signaling) mechanisms (**Figure 3A**) (106). As anti-apoptotic and proliferative signaling are closely linked, many molecules driving proliferation together with mutant p53 also promote chemoresistance. These include p63, p73, KLF17, REG-γ proteosome pathway and PTEN signaling pathway through Bcl-XL (**Figure 3A**) (107–110). Transcriptional de-regulation of certain miRNAs by mutant p53 may also confer chemoresistance. For instance, upregulation of miR-128-2 that targets E2F5 and downregulation of miR-223 which targets *STMN1* confers resistance to chemotherapeutic drugs (**Figure 3A**) (111, 112).

The most extensively studied function of GOF mutant p53 is however its role in promoting invasion and metastasis of cancer cells. Mutant p53 implicate various context and tissue dependent mechanisms to promote cancer cell invasion and metastasis. Mutant 53 can promote invasion and loss of directionality of migration by enhancing integrin and epidermal growth factor receptor (EGFR) trafficking which results in constitutive activation of integrin/EGFR signaling (**Figure 3A**) (113). Importantly, mutant p53 can bind to TAp63 to interfere its function leading to decreased expression of metastasis-inhibiting genes such as Sharp1, CyclinG2, and Dicer (**Figure 3A**) (114, 115). In mutant p53 bearing cells, TGF-β acts in concert with oncogenic Ras to form a complex consisting of mutant-p53-p63 and Smads (114). The formation of this complex inhibits p63 functions and expression of its target genes Sharp1 and Cyclin G2 which are essential mediators of p63-mediated antagonism towards TGFβ signaling (114). Further, mutant p53 inhibits TAp63 mediated transcriptional activation of Dicer leading to an overall depletion of miRNA processing and enhanced metastatic potential (115, 116). Mutant p53 mediated repression of p63 function can also modulate the expression of certain miRNAs involved in invasion and metastasis such as let-7i, miR-155, miR-205, miR-130b, and miR-27a (**Figure 3A**) (117–121). Various transcription factors such as NF-Y, SREBPs, ETS, and EGFR1 play crucial role in mutant p53 driven invasion and metastasis. In pancreatic cancers, mutant p53 activates the NF-Y transcription complex by releasing p73, resulting in transactivation of PDFGR-β (**Figure 3A**), promoting cell migration, while in glioblastoma PTEN promotes the association of mutant p53 with NF-Y to induce expression of Myc and Bcl-XL (110, 122). Mutant p53 in association with NF-Y and p300 can transactivate *EFNB2* to promote EMT *via* Src/Fak signaling (**Figure 3A**) (104). Binding of mutant p53 to ETS2 can promote expression of Pla2g16 or nucleotide synthesis genes required for invasion depending upon the cancer type (**Figure 3A**) (123, 124). Furthermore, the binding of mutant p53 to EGR1 promotes MYO10 expression which drives breast cancer cell invasion (**Figure 3A**) (125). Interaction of mutant p53 to SREBPs activates mevalonate pathway that promotes invasion in breast cancer cells (**Figure 3A**) (126). A recent study by Capaci et al. showed that mutant p53 can interact with HIF1α to induce miR-30d expression which promotes tubulo-vesiculation of Golgi apparatus leading to enhanced vesicular trafficking and secretion (**Figure 3A**) (127). This potentiates the deposition and remodeling of extra-cellular matrix enhancing metastatic colonization and tumorigenesis (127).

One of the important hallmarks of cancer is the process of formation of new blood vessels from existing vasculature or angiogenesis. Mutant p53 promote tumor neo-angiogenesis through the induction of ROS and Hif1-α which induces the expression of pro-angiogenic factor VEGFA (128). Also, the upregulation of ID4 by mutant p53, promotes increased levels of pro-angiogenic cytokines such as IL-8 and Gro-α (129). The increased blood vessel formation in mutant p53 xenografts in comparison to tumors expressing wild type p53, suggests that mutant p53 plays a crucial role in promoting angiogenesis both *in vivo* and *in vitro* (128).

Cancer cells depend on glycolysis to fulfil the energy requirements for continuous growth and proliferation. Several evidence demonstrate that mutant p53 promotes glycolysis and reprograms the cellular metabolism of cancer cells. Zhou et al. showed that mutant p53 binds to novel interacting partner AMPKα in glucose starvation conditions and inhibits its activation by other kinases leading to increased aerobic glycolysis, lipid production, and cell growth (**Figure 3A**) (130). Mutant p53 also increases glucose uptake by triggering translocation of glucose transporter GLUT1 to plasma membrane (131). The increased energy required by the mutant p53 bearing cell during invasion and metastasis is provided by enhanced glycolysis through mutant p53-AMPK binding and mutant p53-SREBP binding which induce expression of mevalonate pathway enzymes (**Figure 3A**) (130, 132). Further, the transcriptional activation of mitochondrial citrate transporter SLC25A1 increases fatty acid and sterol biosynthesis and oxidative phosphorylation (**Figure 3A**) (102).

Mutant p53 promotes the expression of oncogenes such as MYC (110, 133), PCNA (134), KLF17 (108), EGFR (121, 135), and AXL (136), and simultaneously inhibits the function of tumor suppressors like the p53 family proteins, p63 and p73 (107, 137, 138) to sustain continuous proliferation of cancer cells (**Figure 3A**). The ablation of mutant p53 in mouse xenografts resulted in significant reduction of tumor growth suggesting the crucial role of mutant p53 in tumor growth *in vivo* (139). Further, mutant p53 regulation of several nucleotide metabolism genes (NMGs) such as DCK, TK1, TYMS, RRM1/2, and GMPS is required for sustained proliferation and reduced replication stress (**Figure 3A**) (124). Mutant p53 can also promote proliferation by inducing the REG-γ proteosome pathway in association with p300 (**Figure 3A**) (109).

Cancer cells utilize a higher number of replicative origins than normal cells (140). Polostkaia et al. first suggested that DNA replication might be a crucial target of mutant p53 (141). They found that mutant p53 not only upregulates two crucial replication factors, viz. PCNA and MCM2 but also stabilizes their chromatin association in breast cancer cells (**Figure 3A**) (141). A further study reported that mutant p53 enhance the association of mutant p53 and PARP on the replicating DNA (**Figure 3A**) (142). Another report by Datta et al. showed GOF mutant p53 co-operates with an oncogenic transcription factor Myb to transactivate Cdc7 in cancer cells which in turn promote Cdc7/Db4 complex formation leading to increased origin firing (**Figure 3A**) (143). GOF mutant p53 can bind to TopBP1 and attenuate ATR checkpoint response during replication stress (**Figure 3A**) (144). Moreover, it can override the Cdk2 requirement to promote replication by facilitating the interaction between TopBP1 and Treslin (**Figure 3A**) (144). GOF mutant p53 also has been found to inhibit proper restart of stalled or damaged replication forks thus driving genomic instability (145). Mutations in p53 have been associated with dysfunctional checkpoint or altered DNA repair pathways that lead to genomic alteration such as aneuploidy, chromosome translocations and amplifications (**Figure 3A**) (146–148). Mutant p53 also suppress crucial DNA repair proteins such as BRCA1 and RAD17, as a result the cell progresses with the damaged DNA leading to aneuploidy and other genomic alterations (**Figure 3A**) (149). Moreover, mutant p53 has been found to transactivate telomerase maintenance gene hTERT which might be the reason behind altered telomere length and architecture in mutant p53 bearing cells (**Figure 3A**) (146, 150).

Inflammation has been found to promote tumorigenesis by several means and has been characterized as one of the enabling hallmarks of cancer (151, 152). While wild type p53 suppresses inflammatory response by inhibiting the production of cytokines and antagonizing NF-kB activity, mutant p53 on the other hand enhances NF-kB activity in response to TNF-α and promotes inflammation (**Figure 3A**) (152–154). Further, mutant p53 together with c-MAF promote IL1-Ra expression and sustain inflammatory signaling (155). The sustained activation of NF-kB signaling by mutant p53 not only elevate inflammatory response but also protects the cancer cells from cytotoxic effects of tumor microenvironment by activating pro-survival pathways. Mutant p53 can also alter other biological processes to promote oncogenesis. A recent work demonstrated that mutant p53 alters RNA splicing by upregulating the splicing regulator hnRNPK (156). This promotes alteration in GTPase-activating protein (GAPs), the negative regulators of RAS family members, leading to heightened KRAS activity in pancreatic ductal adenocarcinoma (**Figure 3A**) (156).

The stress-responses associated with tumorigenesis represent the common hallmarks of cancer. Mutant p53 support cancer cell survival and proliferation by safeguarding them from the various oncogenic stress and was aptly called “guardian of the cancer cell” (96). These adaptive mechanisms of mutant p53 may explain addiction of cancer cells to mutant p53.

#### Regulation of GOF Mutant p53 by Upstream Signals

The GOF mutant p53 is regulated by various oncogenic stress signals. As mutant p53 lacks the ability to transactivate the ubiquitin ligase MDM2, it was considered that it would be accumulated in both normal and cancer tissues. However, studies with p53 knock in mice shows that its cellular levels vary from being low in normal tissues to high in cancer tissues (157). Different studies have revealed that inherently unstable mutant p53 can be stabilized by genotoxic stress (ionizing radiation, ROS), loss of tumor suppressor proteins (e.g. P16INK4A, PML) and oncogenic insults (Myc, KRas, ErbB2) (158, 159).

Mutant p53 stability and activity are primarily altered by post-translational modifications (PTMs), ubiquitin ligases and specific chaperons (**Figure 3B**). Like wild type p53, GOF mutant p53 can also be post-transcriptionally modified by a variety of genotoxic and cellular stress signals. While these stress signals stabilize wild type p53 to suppress tumorigenesis, they stabilize mutant p53 to exacerbate tumor malignancy. DNA damaging agents such as Gemcitabine has been demonstrated to phosphorylate mutant p53 (R273H) at serine 15 which leads to nuclear accumulation of mutant p53 and increases chemoresistance (160). Chronic S15 phosphorylation of mutant p53 has been found in tumors where DNA damage signaling is constitutively activated (161, 162). Activated Ras signaling promotes phosphorylation of mutant p53(R280K) at S6 and S9, which then associate with Smad2 and TP63 to inhibit the metastasis suppressor function of the latter (114). Further, NF-kB inhibition by overexpression of IkB also results in S15 phosphorylation of mutant p53 *via* GADD45α mediated JNK1 activation (163). Additionally, stathmin1 associated with microtubule dynamics and destabilization, may phosphorylate mutant p53 at S15 and S37 and contribute to its stability (164). DNA damage induced polo-like kinase 2 (PLK2) can also phosphorylate mutant p53 (R175H, R273H) at C terminal serine residue T377, leading to enhanced binding to p300, increased acetylation and GOF activity (165). Mutant p53 acetylation also plays a role in accumulation and GOF activity of mutant p53 (166). According to a report by Minamoto et al., mutant p53 is hyperacetylated at K320, K373, and K382 in multiple cancer cell lines (167). Acetylation of K382 on mutant p53 R273H has also been reported in multiple colon cancer cell lines (168). Jethwa et al. showed that TRRAP, which recruits histone acetyltransferases to chromatin during transcription and DNA repair also stabilize different p53 mutants through inhibition of MDM2-proteasome axis in Burkitt lymphoma (169, 170). On the contrary, Id4 induced interaction of mutant p53 and p300/CBP (P/CAF) promotes acetylation at K320 and K373 resulting in increased expression of p21, BAX, and PUMA leading to apoptosis (171). This suggests that acetylation at K320 and K373 can alter the structure of mutant p53 and restore wild type p53 functions. Mutant p53 stability is also regulated by glucose levels. Glucose deprivation cause deacetylation at C terminal lysine residues and trigger mutant p53 degradation and autophagic cell death (172). Activation of SIRT1 deacetylase by YK-3-237, leads to reduced mutant p53 levels and triggers apoptotic cell death (173). Ubiquitination of mutant p53 also play a crucial role in regulating its stability and subcellular localization. While polyubiquitination of mutant p53 leads to its degradation, monoubiquitination may alter the subcellular localization of mutant p53 affecting its GOF activity (174). DNA damage induced ATM mediated phosphorylation of mutant p53 R175H at S15 results in monoubiquitination by MDM2 instead of polyubiquitination.

Molecular chaperones, such as the heat shock proteins (HSPs) are also known to bind to mutant p53 to refold, stabilize or degrade it (175–177). For example, HSP90 play a crucial role in stabilizing mutant p53. It may form a complex with mutant p53 and MDM2 to block their ubiquitination mediated degradation or may form a complex with mutant p53 to prevent aggregation of mutant p53 by inhibiting MDM2 and CHIP in multiple cancer cell lines (178, 179). Recently, Ingallina et al. showed that mechanical cues such as stiffness of the extracellular matrix trigger RhoA dependent remodeling of actin and actomyosin contractility which leads to mutant p53 accumulation by HDAC6/HSP90 axis (180). HSP70 is also involved in mutant p53 stabilization and degradation (181, 182). HSP70/HSC70 complex can recognize misfolded mutant p53 proteins and promotes its CHIP mediated ubiquitination and degradation when HSP90 activity is inhibited (181). Another member of HSP70 family, mortalin, also binds to mutant p53. Knockdown of mortalin results in nuclear translocation of mutant p53 and triggers apoptosis in HCC cell line, PLC/PRF/5 (183). However, whether mortalin inhibition restores wild type p53 function is not clear. Other than the HSPs, BCL-2 associated anthanogene (BAG) family proteins also interact with mutant p53 to promote its GOF activity by inhibiting ubiquitination mediated degradation by MDM2 and CHIP (184, 185).

Stabilization of mutant p53 promotes its gain-of-function activities. Therefore, disrupting its stability by therapeutically targeting chaperons and other proteins that impart stability to mutant p53 might be beneficial in treatment of aggressive mutant p53 tumors.

#### Impact of GOF Mutations on Cancer Stemness

Enhanced cancer stemness phenotype has emerged as a crucial oncogenic property of mutant p53 in recent years. The novel *gain-of-function* property of mutant p53 to enhance somatic cell reprogramming efficiency was first proposed by Sarig *et al.* in 2010 (186). They showed that GOF-mutant p53 bearing mouse embryonic fibroblasts (MEFs) reprogrammed more efficiently than p53 knockout MEFs (186). This indicates that GOF mutant p53 not only prevent elimination of sub-optimized cells by apoptosis but also facilitate in acquisition of pluripotency. Furthermore, while reprogrammed cells with p53 deficiency formed differentiated teratomas *in vivo*, those with GOF mutant p53 formed undifferentiated malignant tumors, implying that it confers oncogenic properties to the reprogrammed cells (186). A few years later, Grespi *et al.* identified a set of miRNAs whose expression altered in a p53-dependent manner during transition of mouse embryonic fibroblasts to induced pluripotent stem cells (187). The role of these miRNAs can further be investigated to determine their role in regulation of mutant p53 driven stemness. A recent study by Solomon et al. propounded that mutant p53 expressing colorectal cancer cell lines harbor an increased population of CD44, Lgr5, and ALDH positive cancer stem cells (15). Further experimental evidences showed that mutant p53 transcriptionally upregulates these CSC markers to promote cancer stem cell population in colorectal cancer cells (**Figure 2**) (15). In another study, Escoll et al. proposed that GOF mutant p53 promotes cancer stemness in glioblastoma and breast cancer cells by activating PI3K/AKT2-mediated integrin or growth factor (GF) receptor cycling. This promotes phosphorylation of WASP-interacting protein (WIP) by AKT2 which in turn stabilizes YAP/TAZ, and supports cancer stem cell survival and phenotypic maintenance (**Figure 2**) (14). Mutant p53 can also induce YAP/TAZ nuclear localization by interacting with SREBP and activating the mevalonate pathway (188). The mevalonate cascade produces geranylgeranyl pyrophosphate which activates Rho-GTPases that in turn activate YAP/TAZ and promotes self-renewal of breast cancer cells (188). Apart from these discrete studies, the molecular mechanism of mutant p53 mediated stemness phenotype is largely unexplored. As cancer stem cell phenotype is extensively driven by epigenetic factors, especially miRNAs, it would be interesting to investigate the GOF mutant p53 altered miRNAs for their possible role in stemness (189).

The major oncogenic properties of enhanced metastasis, chemoresistance and angiogenesis conferred by GOF mutant p53 are also integral to cancer stem cells. Hence, understanding the molecular and phenotypic characteristics common to CSCs and GOF mutant p53 cells might unravel new mechanisms by which these p53 mutants promote stem-like phenotype in cancer cells.

##### Association With EMT

During development, embryonic cells possessing high degree of cellular plasticity undergo reversible transformations and migrate long distances to form tissues and organs. To facilitate migration, the epithelial cells acquire mesenchymal characteristics by a process known as epithelial to mesenchymal transition (EMT). Upon reaching their destination, they revert to epithelial phenotype by the process of mesenchymal to epithelial transition (MET) to settle, proliferate, and differentiate into different organs (190). These key developmental programs are often reactivated in cancer cells which lead to cancer invasion and metastasis. However, unlike in embryogenesis, EMT associated with cancer involves intravasation of delaminated cells into blood and lymphatic vessels and subsequent extravasation to colonize at distant sites. EMT is triggered by many extracellular signals and agents such as members of the transforming growth factor β (TGF-β)/bone morphogenetic protein (BMP) family, Wnt, Notch, epidermal growth factor, fibroblast growth factor, hypoxia, UV light, nicotine, and many others (191). Such signals stimulate the activation of certain transcription factors (TFs) such as Snail, Twist, Zeb, and others which may act independently or in combination to suppress epithelial phenotype and enhance mesenchymal traits such as motility, ability to degrade basement membrane and extracellular matrix (192, 193).

Metastasis involves two phases, the first involves dissemination of cancer cells from the primary site and translocation to a distant organ and second, the ability of the cancer cells to develop a tumor at the secondary site (194). At both the levels the critical role of CSCs is obvious. Primarily, the ability of the disseminated cells to seed secondary tumor and differentiate into non-stem cells are the very traits of self-renewal and tumor-initiating ability, that define CSCs. The migrating cancer cells also exhibit other features of CSCs, namely cell motility, invasiveness, and increased chemoresistance (194). Brabletz et al. termed the metastasizing cell population bearing stemness features as “migratory cancer stem cells” and proposed that they arise from stationary cancer stem cells through the gain of EMT phenotype (195). On the contrary, Chauffer et al. proposed that the presence of two CSC population in tumor; the intrinsic CSCs that are inherently present in the tumor and induced CSCs that arise from differentiated tumor cells as a consequence of EMT signaling (194). There are several reports of acquisition of stem-like features in cancer cells upon induction of EMT. Mani et al. found that induction of EMT trigger expression of stem cell markers in addition to acquisition of mesenchymal traits (196). Furthermore, the cells undergoing EMT exhibited similar mammosphere forming ability as the stem cells isolated from culture. Similarly, Morel et al. reported that EMT induction accelerate the transition of CD44^low^CD24^+^ cells to CD44^+^CD24^-^ cells through the activation Ras/MAPK signaling (197).

One of the major gain-of-function properties of mutant p53 is invasion and metastasis. However, whether mutant p53 induced EMT trigger stemness properties in cancer cells, is still quite unexplored. Wild type p53 promotes epithelial differentiation through transcriptional activation of miR-200c (198) which inhibit the translation of EMT activator Zeb1 (**Figure 2**) (199, 200). Zeb1 and Zeb2 in turn repress the other miRNAs of miR-200c family that targets self-renewal factors like Bmi1 (201), and possibly Klf4 and Sox2. Therefore, loss of p53 in mammary epithelial cells leads to a reduced expression of miR-200c thereby promoting EMT and stemness properties and development of a high-grade tumor (198). These observations were corroborated by Pinho *et al.* study in pancreatic acinar cells where they found that loss of p53 leads to increased levels of stemness regulators Bmi1 and Klf4, as well as Vimentin and EMT inducers such as, Snail, Twist, Zeb1, and Zeb2 (88). Although, they did not find any connecting link between the increased stemness and enhanced epithelial to mesenchymal transition phenotype displayed by the p53−/− cells, a high expression of miR-200c can be assumed to be the underlying cause. *TP53* has also been implicated in the suppression of EMT and stemness in the PC-3 prostate cancer cells by modulating the expression of miR-145 (202). PC3 cells expressing wild type p53 were found to express high levels of epithelial marker E-cadherin while the expression of mesenchymal markers fibronectin, vimentin, N-cadherin, and Zeb2 as well as CSC markers such as CD44, Oct4, c-Myc, and Klf4 were reduced. This was rescued upon inhibition of miR-145 in those cells (202). Taken together, *TP53* plays a crucial role in maintaining epithelial phenotype and suppresses pluripotency factors to maintain a differentiated state. However, with the loss of p53 function the suppression on pluripotency genes is lost and this results in activation of EMT and stemness factors. Gain-of function mutant p53 further promotes EMT and stemness phenotypes by activating genes regulating them. For example, in a study by Dong et al., mutant p53 was found to suppress miR-130b expression by binding to its promoter, thereby upregulating the expression of Zeb1, the downstream target of miR-130b (**Figure 2**) (120). Activation of Zeb1 signaling induce Bmi1 expression and promotes stemness (**Figure 2**) (120). Another wild type p53 responsive miRNA, miR-194 has been found to be negatively regulated by mutant p53 in endometrial cancer cells. As miR-194 targets the oncogene Bmi1 which mediates pluripotency, suppression of this miRNA by mutant p53 leads to cancer stemness and EMT phenotypes (**Figure 2**) (120). Mutant p53-R273H has also been found to upregulate lncRNAs, lnc273-31, and lnc273-34 implicated in EMT and CSC maintenance in colorectal cancer cells (**Figure 2**) (203). Although these studies highlight that mutant p53 mediated EMT phenotype confer stemness in cancer cells, however, there is still a lot to explore in context of molecular mechanisms of mutant p53 driven stemness through activation of EMT genes.

##### Association With Chemoresistance

One of the major oncogenic gain-of-functions conferred by mutant p53 to the cancer cells is chemoresistance. Mutant p53 singularly regulate a number of pivotal pathways, all of which promote resistance to chemotherapeutic drugs. It is interesting to note that the specific pathways altered by mutant p53 to confer chemoresistance are central to the drug-resistance ability of the CSCs. For example, CSCs abundantly express ABC transporters, that exports drugs out of the cells and imparts chemoresistance (204). Interestingly, one of the important proteins of the ABC family, MDR1, that remains suppressed by wild type p53 in normal cells, is stimulated by mutant p53 in cancer cells during tumorigenesis (**Figure 2**) (80). When normal cells encounter drug induced DNA damage, p53 is stabilized and it triggers cell death by apoptosis. This function is completely lost in mutant p53 cells. In addition, GOF mutant p53 augment the expression of anti-apoptotic proteins Bcl-2 and Bcl-x_L_ and repress pro-apoptotic proteins Bax, Bad, and Bid (**Figure 2**) (205). In a similar manner, CSCs suppress the Bcl-2 family proteins to attenuate drug-induced cell death (206). DNA-repair mechanisms are mostly impaired in somatic cancer cells. However, CSCs express high levels of DNA-repair genes that helps them repair DNA damage inflicted by chemotherapeutic drugs (207). Murine mesenchymal stem cells (MSCs) with p53 mutations were also found to express high levels homologous recombination repair and non-homologous end joining genes like CSCs (208). Also, mutant p53 expressing iPSCs that induce aggressive tumor in mice, express high levels of detoxifying enzyme associated with drug resistance (15). Despite these similarities there are not many reports on role of CSCs in drug resistance of mutant p53 cells except some indirect ones.

##### Association With Inflammation and Angiogenesis

GOF mutant p53 can modify the tumor microenvironment and has been found to support chronic inflammation (154). Cancer associated p53 mutants elevate NF-kB activity in response to the cytokine TNF-α and drives cancer progression by elevating inflammatory response (209). Inflammatory response triggered by cytokines has been demonstrated to cause dedifferentiation of cancer cells to CSCs through the activation of various signaling pathways including NF-kB signaling pathway (210). Therefore, it may be presumed that immune response in GOF mutant p53 cells drives cancer stemness by activation of NF-kB pathway. CSCs also exhibit the prominent *gain-of-function* property to induce angiogenesis. Mutant p53 promotes the formation of new blood vessels in tumor by regulating the pro-angiogenic factor VEGF (128). The cancer stem cell niche which supports the long term growth of CSCs, secrete factors that stimulate angiogenesis (211). Moreover, stem cell-like glioma cells (SCLGC) have been found to elevate VEGF to promote angiogenesis (212).

Therefore, it can be surmised that mutant p53 mediated oncogenic *gain-of-functions* potentially drives dedifferentiation of cancer cells to cancer stem cells and *vice-versa* and underlies the enhanced tumorigenesis and poor prognosis of human cancers with p53 mutations.

## Prospective Therapeutic Approaches Targeting CSCs in Mutant p53 Tumors

Cancer stem cells can arise either from mutations in normal stem/progenitor cells or dedifferentiation of cancer cells (151, 213). Irrespective of the origin, CSCs feature quiescence, ability of self-renewal, therapeutic resistance and metastatic potential (214–216). Loss of wild type p53 function and simultaneous gain of new oncogenic functions by certain missense mutant p53 can generate CSCs or CSC-like features (84, 90, 217–220). Therapeutics that target the intersection between modalities of CSC and p53 mutations are the focus of this section. Many of the discussed therapeutic interventions relevant for targeting CSCs are already in clinical trials in the context of treating mutant p53-based adversities (**Table 1**). Other approaches in restoring wild type p53 functions have been detailed elsewhere (220, 221).

**Table 1:** Some clinical trials targeting common mechanistic pathways related to both mutant p53 and cancer stem cells.

Product name	Pathways involved	Phase	Status	Clinical trial registration	Link
Statin	mevalonate pathway	Phase 2	recruiting	NCT03358017	clinicaltrials.gov/ct2/show/NCT03358017
Metformin	mTOR pathway	Phase 1	completed	NCT01981525	clinicaltrials.gov/ct2/show/NCT01981525
		Phase 2	recruiting	NCT03047837	clinicaltrials.gov/ct2/show/NCT03047837
		Phase 1	completed	NCT02312661	clinicaltrials.gov/ct2/show/NCT02312661
SAHA or vorinostat	proteasomal degradation	Phase 1	active, non-recruiting	NCT02042989	clinicaltrials.gov/ct2/show/NCT02042989
AZD1775 or MK1775	cell cycle regulation	Phase 2	completed	NCT01357161	clinicaltrials.gov/ct2/show/NCT01357161
		Phase 2	active, non-recruiting	NCT02101775	clinicaltrials.gov/ct2/show/NCT02101775
		Early Phase 1	recruiting	NCT02659241	clinicaltrials.gov/ct2/show/NCT02659241

### Targeting the Hallmarks of Cancer Pronounced in CSC and p53 Mutant Tumor Cells

Certain hallmarks of cancer like invasion, modified metabolism and proliferation have been found to be active in CSCs as well as p53 mutant tumor cells. Mutant p53 activates SREBP target genes inducing mevalonate pathway that drives cancer cell reprogramming. Mevalonate pathway is lipogenic yielding isoprenoids and cholesterol. Isoprenoids carry out protein prenylation/lipidation and enables proteins like Ras and Rho GTPases to attach with the cell membrane (222). YAP/TAZ, that works through Hippo signaling pathway, induce tissue regeneration, disorganized polarity, CSC features like chemoresistance and metastasis (223–225). YAP/TAZ, together with mutant p53 and NFY transactivate cyclin A, cyclin B and CDK1 promoting cancer growth (226). A functional association among mevalonate enzymes, mutant p53, Rho GTPases, and YAP/TAZ has been implicated (180, 219). SREBP-mevalonate axis is relevant for YAP/TAZ mediated tumor progression. Cholesterol-lowering drug, statins, inhibits HMG-CoA reductase of mevalonate pathway, and blunt YAP/TAZ mediated growth of mutant p53-bearing tumors (**Table 1**) (**Figure 3**) (188). Another instance of metabolic rewiring is the ability of mutant p53 to restrain autophagy by inhibiting AMPK and inducing mTOR pathway thereby ensuring tumor growth (227). In absence of AMPK, mitochondrial stress augments aerobic glycolysis, also called “Warburg effect” in tumor cells, which is promoted by mutant p53 (131). This is potentiated by its tendency of higher glucose uptake aided by mutant p53-mediated increased translocation of glucose transporter GLUT1 to cell membrane (131). Warburg effect is one of the striking features altered metabolism in CSCs (228, 229). Treatment with antidiabetic drug, metformin (**Figure 3**), and mTOR inhibitor, everolimus, has shown to reduce tumor growth and are being tested in clinical trials (**Table 1**) (230).

In breast cancer cells and mutant p53-KI mouse model of Li-Fraumeni Syndrome, phosphorylation-dependent prolyl-isomerase, Pin1, has shown to augment mutant p53 GOF activities including cellular migration and invasion marked as CSC properties (231, 232). All-trans retinoic acid (ATRA), used in acute promyelocytic leukemia, binds and degrades Pin1 (**Figure 3**) (233). MRX34 is a mimic of miR-34, which can restore the lost tumor suppressor function of mutant p53 (234). Wild type p53 induces miR-34 that can inhibit both tumorigenesis and reprogramming by suppressing myriad genes like like cyclin D1, cyclin E2, CDK4, and CDK6 involved in proliferation; Nanog, N-Myc, SOX2 involved in pluripotency and, SNAIL involved in EMT (235, 236). The phase I study on MRX34 has been recently reported (237). Linc-RNA SOX21-ASI and Linc-RNA HOTAIR can also be important targets as they regulate miR-429 and miR-34a expressions to maintain CSC phenotype (238). Cells bearing mutant p53 depend on G2-M check point for DNA repair, which results from WEE-1 mediated phosphorylation of Tyr15 of Cdk1, inactivating the Cdk1/CyclinB complex required for G2 to M progression (239). WEE-1 inactivation abrogates G2-M checkpoint and drives cells into unscheduled mitosis and death by mitotic lethality (240). The WEE-1 inhibitor, AZD1775 (MK1775), has been included in several clinical trials (**Table 1**) (219). It has been recently found to target CSC properties in breast cancer (241).

### p53 Family—An Important Aspect in CSC Regulation

A gain-of-function property of mutant p53 is ability to complex with its family proteins, p63 and p73, which however are not frequently mutated in cancers (242). p53 family members and their isoforms have contrasting effects on differentiation. Wild type p53 and p73 induces differentiation whereas, p63 drives epithelial stem cell proliferation (215, 220). On the other hand, ΔNp73 and ΔNp63 induces enrichment of CSC characters (220, 243, 244). p63 and p73 also play anti-metastatic and pro-apoptotic roles, respectively (114, 245). Mutant p53 can itself disrupt the balance between stem cell proliferation and differentiation as well as sequester p63 or p73 thereby hindering apoptosis, augmenting proliferation, and driving chemoresistance and metastasis typical of cancer stem cells (9, 246–248). Mutant p53–p63 complex can increase RAB coupling protein (RCP)-mediated recycling of cell surface growth-promoting receptors (249). Ras-dependent phosphorylation at Ser6 and Ser9 of mutant p53 forms mtp53-SMAD complex that inhibits p63-mediated anti-metastatic effect (250). Hence, p53 family members present a larger scope of targeting mutant p53-mediated oncogenicity in the context of CSC.

The compound, RETRA disrupts mutant p53-p73 complex restoring p73-dependent transcription and apoptosis (**Figure 3**) (251). Other compounds known to restore effects of wild type p53 in a p73-dependent manner are NSC176327, NSC143491, NSC254681, mTOR inhibitor rapamycin, NSC59984, and prodigiosin (252–254). Short Interfering Mutant p53 Peptides (SIMP) can interact with different mutant p53 proteins and release p73, while peptides aptamers (PA) can inhibit mutant p53 transcription (**Figure 3**) (255).

### Therapeutics to Destabilize Mutant p53

Wild type p53 undergoes proteasomal degradation with the help of E3 ubiquitin ligase, MDM2, which in turn is transactivated by wild type p53. However, mutant p53 is unable to transcribe MDM2 causing its cellular stabilization, which is essential for its GOF manifestation (256). Moreover, heat shock protein HSP90 chaperone machinery prevents mutant p53 ubiquitylation and fosters chemoresistance, which is an intrinsic property of CSC (139). Hsp90 stabilizes mutant p53 by inactivating E3 ubiquitin ligases, DM2 and CHIP (257).

Hsp90 can be inhibited by 17AAG or its derivative, 17DMAG, in combination with HDAC inhibitor suberoylanilide hydroxamic acid (SAHA/vorinostat) (**Table 1**, **Figure 3**) (257). Ganetespib is another Hsp90 inhibitor used in similar context (139). Panaxynol is another Hsp90 inhibitor that reportedly targets lung cancer stem cells (258). Bortezomib and carfilzomib are FDA-approved proteasomal inhibitors for treating multiple myeloma (259). However, mutant p53 in cooperation with Nrf2 transactivates proteasome thereby raising resistance in triple negative breast cancer (260). The resistance can be overcome by combination therapy with APR-246, a molecule that can restore native p53 conformation in GOF mutant p53 (221, 260). Stabilization of Nrf2, which regulates cellular antioxidation, has also been linked to chemoresistance in the context of CSC (261).

### Poly (ADP Ribose) Polymerase Inhibition—An Elusive Promise?

Mutant p53 sequesters MRE11 hindering ATM-mediated double strand break repair (161, 262). It can complex with E2F4 and downregulate homologous recombination factor, BRCA1 and, single strand break repair factor, Rad17 (149). However, it potentiates the replication factors, topoisomerase 1 (Top1), PCNA and MCM4, and the error-prone repair factor, poly(ADP ribose) polymerase 1 (PARP1) (141, 263). This underscores the significance of PARP1 inhibitors (PARPi) to augment synthetic lethality in the context of mutant p53-mediated incapacitation of DNA repair (**Figure 3**) (141, 264). PARPi has been found to induce chemosensitivity in colorectal cancer stem cells (265). However, similar therapy has shown to enrich resistant CD133^+^ ovarian CSCs by inducing alternative DNA repair based on DNA meiotic recombinase 1 (DMC1) (266).

## Concluding Remarks

Stem cells residing at the apex of tissue hierarchy, self-renew and differentiate to maintain tissue homeostasis and ensure proper development and regeneration. Imbalance between these two processes results in tissue malfunction and formation of tumor. p53 plays a crucial role in maintaining this balance and conserves tissue hierarchy. It also acts a barrier for dedifferentiation and reprogramming and prevents the transformation of somatic cells to stem cells. In response to DNA damage, activated p53 either promotes differentiation or triggers apoptosis, thereby preserving genome integrity of SCs. Loss or *gain-of-function* mutations in *TP53* induce dedifferentiation and proliferation of SCs with damaged DNA leading to the generation of CSCs.

GOF mutant p53 augments malignant transformation by promoting cell proliferation, metastasis, angiogenesis, resistance to cell death and chemotherapeutic drugs. In recent years, GOF mutant p53 has been implicated in promoting somatic cell reprogramming, CSCs formation and expansion. CSCs, the cornerstone for tumor initiation, progression, and relapse share several oncogenic properties with GOF mutant p53 cells. Therefore, it would be interesting to investigate whether these oncogenic phenotypes are conferred by the increased CSC population residing in GOF mutant p53 tumors or *vice-versa*. As CSCs contribute to drug-resistance and subsequent tumor relapse, targeting them may improve the therapeutic efficacy in *TP53*-mutated tumors. Conceptually, drugs that target common pathways operating in mutant p53 cells and CSCs might have better therapeutic efficacy than those that solely target mutant p53. A few such drugs are already in different phases of clinical trial. Further insights into the underlying molecular mechanisms of mutant p53-driven heightened stemness can open up new therapeutic avenues to selectively target aggressive CSCs in *TP53*-mutated human cancers.

## Author Contributions

The manuscript was designed and conceptualized by DG, DDG, and SR. DG and DDG contributed to the section on wild type p53 and stemness. DG contributed to the section on the effect of p53 inactivation and p53 GOF mutations on stemness. DDG contributed to the section on therapeutic strategies. DG prepared the figures. The manuscript was critically revised by SR. All authors contributed to the article and approved the submitted version.

## Funding

The work was supported by J.C. Bose National Fellowship grant, JCB/2017/000005 awarded to SR and Women Scientist Grant, SR/WOS-A/LS-42/2017 awarded to DDG.

## Conflict of Interest

The authors declare that the research was conducted in the absence of any commercial or financial relationships that could be construed as a potential conflict of interest.

## References

[1] Chen J The Cell-Cycle Arrest and Apoptotic Functions of p53 in Tumor Initiation and Progression. Cold Spring Harbor Perspect Med (2016) 6:a026104–a026104. 10.1101/cshperspect.a026104 PMC4772082 26931810

[2] Koifman G Aloni-Grinstein R Rotter V p53 balances between tissue hierarchy and anarchy. J Mol Cell Biol (2019) 11:553–63. 10.1093/jmcb/mjz022 PMC6735948 30925590

[3] Olivier M Hollstein M Hainaut P TP53 mutations in human cancers: origins, consequences, and clinical use. Cold Spring Harbor Perspect Biol (2010) 2:a001008–a001008. 10.1101/cshperspect.a001008 PMC2827900 20182602

[4] Freed-Pastor WA Prives C Mutant p53: one name, many proteins. Genes Dev (2012) 26:1268–86. 10.1101/gad.190678.112 PMC3387655 22713868

[5] Sabapathy K Lane DP Therapeutic targeting of p53: all mutants are equal, but some mutants are more equal than others. Nat Rev Clin Oncol (2018) 15:13–30. 10.1038/nrclinonc.2017.151 28948977

[6] Brosh R Rotter V When mutants gain new powers: news from the mutant p53 field. Nat Rev Cancer (2009) 9:701–13. 10.1038/nrc2693 19693097

[7] Lozano G The oncogenic roles of p53 mutants in mouse models. Curr Opin Genet Dev (2007) 17:66–70. 10.1016/j.gde.2006.12.003 17208429

[8] Oren M Rotter V Mutant p53 gain-of-function in cancer. Cold Spring Harbor Perspect Biol (2010) 2:a001107–a001107. 10.1101/cshperspect.a001107 PMC2828285 20182618

[9] Strano S Dell’Orso S Di Agostino S Fontemaggi G Sacchi A Blandino G Mutant p53: an oncogenic transcription factor. Oncogene (2007) 26:2212–9. 10.1038/sj.onc.1210296 17401430

[10] Aschauer L Muller PAJ Novel targets and interaction partners of mutant p53 Gain-Of-Function. Biochem Soc Trans (2016) 44:460–6. 10.1042/BST20150261 27068955

[11] Donghi R Longoni A Pilotti S Michieli P Della Porta G Pierotti MA Gene p53 mutations are restricted to poorly differentiated and undifferentiated carcinomas of the thyroid gland. J Clin Invest (1993) 91:1753–60. 10.1172/JCI116385 PMC288155 8473515

[12] Ben-Porath I Thomson MW Carey VJ Ge R Bell GW Regev A An embryonic stem cell-like gene expression signature in poorly differentiated aggressive human tumors. Nat Genet (2008) 40:499–507. 10.1038/ng.127 18443585 PMC2912221

[13] Robles AI Harris CC Clinical outcomes and correlates of TP53 mutations and cancer. Cold Spring Harb Perspect Biol (2010) 2:a001016–a001016. 10.1101/cshperspect.a001016 20300207 PMC2829964

[14] Escoll M Gargini R Cuadrado A Anton IM Wandosell F Mutant p53 oncogenic functions in cancer stem cells are regulated by WIP through YAP/TAZ. Oncogene (2017) 36:3515–27. 10.1038/onc.2016.518 28166194

[15] Solomon H Dinowitz N Pateras IS Cooks T Shetzer Y Molchadsky A Mutant p53 gain of function underlies high expression levels of colorectal cancer stem cells markers. Oncogene (2018) 37:1669–84. 10.1038/s41388-017-0060-8 PMC6448595 29343849

[16] Reya T Morrison SJ Clarke MF Weissman IL Stem cells, cancer, and cancer stem cells. Nature (2001) 414:105–11. 10.1038/35102167 11689955

[17] Visvader JE Lindeman GJ Cancer stem cells in solid tumours: accumulating evidence and unresolved questions. Nat Rev Cancer (2008) 8:755–68. 10.1038/nrc2499 18784658

[18] Nowell PC The clonal evolution of tumor cell populations. Sci (New York NY) (1976) 194:23–8. 10.1126/science.959840 959840

[19] Visvader JE Lindeman GJ Cancer Stem Cells: Current Status and Evolving Complexities. Cell Stem Cell (2012) 10:717–28. 10.1016/j.stem.2012.05.007 22704512

[20] Batlle E Clevers H Cancer stem cells revisited. Nat Med (2017) 23:1124–34. 10.1038/nm.4409 28985214

[21] Pattabiraman DR Weinberg RA Tackling the cancer stem cells - what challenges do they pose? Nat Rev Drug Discovery (2014) 13:497–512. 10.1038/nrd4253 24981363 PMC4234172

[22] Lopez-Garcia C Klein AM Simons BD Winton DJ Intestinal stem cell replacement follows a pattern of neutral drift. Sci (New York NY) (2010) 330:822–5. 10.1126/science.1196236 20929733

[23] Snippert HJ van der Flier LG Sato T van Es JH van den Born M Kroon-Veenboer C Intestinal crypt homeostasis results from neutral competition between symmetrically dividing Lgr5 stem cells. Cell (2010) 143:134–44. 10.1016/j.cell.2010.09.016 20887898

[24] Stanger BZ Cellular homeostasis and repair in the mammalian liver. Annu Rev Physiol (2015) 77:179–200. 10.1146/annurev-physiol-021113-170255 25668020 PMC5830102

[25] Plaks V Kong N Werb Z The cancer stem cell niche: how essential is the niche in regulating stemness of tumor cells? Cell Stem Cell (2015) 16:225–38. 10.1016/j.stem.2015.02.015 PMC4355577 25748930

[26] Rich JN Cancer stem cells: understanding tumor hierarchy and heterogeneity. Medicine (2016) 95:S2–7. 10.1097/MD.0000000000004764 PMC5599210 27611934

[27] Wiechert A Saygin C Thiagarajan PS Rao VS Hale JS Gupta N Cisplatin induces stemness in ovarian cancer. Oncotarget (2016) 7:30511–22. 10.18632/oncotarget.8852 PMC5058697 27105520

[28] Schwitalla S Fingerle AA Cammareri P Nebelsiek T Göktuna SI Ziegler PK Intestinal tumorigenesis initiated by dedifferentiation and acquisition of stem-cell-like properties. Cell (2013) 152:25–38. 10.1016/j.cell.2012.12.012 23273993

[29] Westphalen CB Asfaha S Hayakawa Y Takemoto Y Lukin DJ Nuber AH Long-lived intestinal tuft cells serve as colon cancer-initiating cells. J Clin Invest (2014) 124:1283–95. 10.1172/JCI73434 PMC3934168 24487592

[30] Gupta PB Fillmore CM Jiang G Shapira SD Tao K Kuperwasser C Stochastic State Transitions Give Rise to Phenotypic Equilibrium in Populations of Cancer Cells. Cell (2011) 146:633–44. 10.1016/j.cell.2011.07.026 21854987

[31] Shimokawa M Ohta Y Nishikori S Matano M Takano A Fujii M Visualization and targeting of LGR5(+) human colon cancer stem cells. Nature (2017) 545:187–92. 10.1038/nature22081 28355176

[32] de Sousa e Melo F Kurtova AV Harnoss JM Kljavin N Hoeck JD Hung J A distinct role for Lgr5(+) stem cells in primary and metastatic colon cancer. Nature (2017) 543:676–80. 10.1038/nature21713 28358093

[33] Chen J Li Y Yu T-S McKay RM Burns DK Kernie SG A restricted cell population propagates glioblastoma growth after chemotherapy. Nature (2012) 488:522–6. 10.1038/nature11287 PMC3427400 22854781

[34] Jain AK Allton K Iacovino M Mahen E Milczarek RJ Zwaka TP p53 Regulates Cell Cycle and MicroRNAs to Promote Differentiation of Human Embryonic Stem Cells. PloS Biol (2012) 10:e1001268. 10.1371/journal.pbio.1001268 22389628 PMC3289600

[35] Maimets T Neganova I Armstrong L Lako M Activation of p53 by nutlin leads to rapid differentiation of human embryonic stem cells. Oncogene (2008) 27:5277–87. 10.1038/onc.2008.166 18521083

[36] Zhang ZN Chung SK Xu Z Xu Y Oct4 maintains the pluripotency of human embryonic stem cells by inactivating p53 through Sirt1-mediated deacetylation. Stem Cells (2014) 32:157–65. 10.1002/stem.1532 PMC3947311 24038750

[37] Schmid P Lorenz A Hameister H Montenarh M Expression of p53 during mouse embryogenesis. Development (1991) 113:857–65. 10.1242/dev.113.3.857 1821855

[38] Han MK Song EK Guo Y Ou X Mantel C Broxmeyer HE SIRT1 regulates apoptosis and Nanog expression in mouse embryonic stem cells by controlling p53 subcellular localization. Cell Stem Cell (2008) 2:241–51. 10.1016/j.stem.2008.01.002 PMC2819008 18371449

[39] Lee DF Su J Ang YS Carvajal-Vergara X Mulero-Navarro S Pereira CF Regulation of embryonic and induced pluripotency by aurora kinase-p53 signaling. Cell Stem Cell (2012) 11:179–94. 10.1016/j.stem.2012.05.020 PMC3413175 22862944

[40] Sabapathy K Klemm M Jaenisch R Wagner EF Regulation of ES cell differentiation by functional and conformational modulation of p53. EMBO J (1997) 16:6217–29. 10.1093/emboj/16.20.6217 PMC1326306 9321401

[41] Lin T Lin Y p53 switches off pluripotency on differentiation. Stem Cell Res Ther (2017) 8:44. 10.1186/s13287-017-0498-1 28241890 PMC5330084

[42] Qin H Yu T Qing T Liu Y Zhao Y Cai J Regulation of apoptosis and differentiation by p53 in human embryonic stem cells. J Biol Chem (2007) 282:5842–52. 10.1074/jbc.M610464200 17179143

[43] Solozobova V Blattner C p53 in stem cells. World J Biol Chem (2011) 2:202–14. 10.4331/wjbc.v2.i9.202 PMC3178757 21949570

[44] Grandela C Pera MF Wolvetang EJ p53 is required for etoposide-induced apoptosis of human embryonic stem cells. Stem Cell Res (2008) 1:116–28. 10.1016/j.scr.2007.10.003 19383392

[45] Lin T Chao C Saito SI Mazur SJ Murphy ME Appella E p53 induces differentiation of mouse embryonic stem cells by suppressing Nanog expression. Nat Cell Biol (2005) 7:165–71. 10.1038/ncb1211 15619621

[46] Akdemir KC Jain AK Allton K Aronow B Xu X Cooney AJ Genome-wide profiling reveals stimulus-specific functions of p53 during differentiation and DNA damage of human embryonic stem cells. Nucleic Acids Res (2014) 42:205–23. 10.1093/nar/gkt866 PMC3874181 24078252

[47] Lee KH Li M Michalowski AM Zhang X Liao H Chen L A genomewide study identifies the Wnt signaling pathway as a major target of p53 in murine embryonic stem cells. Proc Natl Acad Sci USA (2010) 107:69–74. 10.1073/pnas.0909734107 20018659 PMC2806696

[48] Ungewitter E Scrable H Delta40p53 controls the switch from pluripotency to differentiation by regulating IGF signaling in ESCs. Genes Dev (2010) 24:2408–19. 10.1101/gad.1987810 PMC2964751 21041409

[49] Donehower LA Harvey M Slagle BL McArthur MJ Montgomery CA Butel JS Mice deficient for p53 are developmentally normal but susceptible to spontaneous tumours. Nature (1992) 356:215–21. 10.1038/356215a0 1552940

[50] Rotter V Schwartz D Almon E Goldfinger N Kapon A Meshorer A Mice with reduced levels of p53 protein exhibit the testicular giant-cell degenerative syndrome. Proc Natl Acad Sci USA (1993) 90:9075–9. 10.1073/pnas.90.19.9075 PMC47504 8415656

[51] Hu W Feng Z Teresky AK Levine AJ p53 regulates maternal reproduction through LIF. Nature (2007) 450:721–4. 10.1038/nature05993 18046411

[52] Takahashi K Tanabe K Ohnuki M Narita M Ichisaka T Tomoda K Induction of pluripotent stem cells from adult human fibroblasts by defined factors. Cell (2007) 131:861–72. 10.1016/j.cell.2007.11.019 18035408

[53] Takahashi K Yamanaka S Induction of pluripotent stem cells from mouse embryonic and adult fibroblast cultures by defined factors. Cell (2006) 126:663–76. 10.1016/j.cell.2006.07.024 16904174

[54] Yamanaka S Strategies and new developments in the generation of patient-specific pluripotent stem cells. Cell Stem Cell (2007) 1:39–49. 10.1016/j.stem.2007.05.012 18371333

[55] Zhao Y Yin X Qin H Zhu F Liu H Yang W Two Supporting Factors Greatly Improve the Efficiency of Human iPSC Generation. Cell Stem Cell (2008) 3:475–9. 10.1016/j.stem.2008.10.002 18983962

[56] Kawamura T Suzuki J Wang YV Menendez S Morera LB Raya A Linking the p53 tumour suppressor pathway to somatic cell reprogramming. Nature (2009) 460:1140–4. 10.1038/nature08311 PMC2735889 19668186

[57] Hong H Takahashi K Ichisaka T Aoi T Kanagawa O Nakagawa M Suppression of induced pluripotent stem cell generation by the p53-p21 pathway. Nature (2009) 460:1132–5. 10.1038/nature08235 PMC2917235 19668191

[58] Marión RM Strati K Li H Murga M Blanco R Ortega S A p53-mediated DNA damage response limits reprogramming to ensure iPS cell genomic integrity. Nature (2009) 460:1149–53. 10.1038/nature08287 PMC3624089 19668189

[59] Li Y Feng H Gu H Lewis DW Yuan Y Zhang L The p53–PUMA axis suppresses iPSC generation. Nat Commun (2013) 4:2174. 10.1038/ncomms3174 23873265 PMC4394110

[60] Bao X Wu H Zhu X Guo X Hutchins AP Luo Z The p53-induced lincRNA-p21 derails somatic cell reprogramming by sustaining H3K9me3 and CpG methylation at pluripotency gene promoters. Cell Res (2015) 25:80–92. 10.1038/cr.2014.165 25512341 PMC4650593

[61] Wang J He Q Han C Gu H Jin L Li Q p53-facilitated miR-199a-3p regulates somatic cell reprogramming. Stem Cells (2012) 30:1405–13. 10.1002/stem.1121 22553189

[62] Yamanaka S A Fresh Look at iPS Cells. Cell (2009) 137:13–7. 10.1016/j.cell.2009.03.034 19345179

[63] Zheng H Ying H Yan H Kimmelman AC Hiller DJ Chen AJ p53 and Pten control neural and glioma stem/progenitor cell renewal and differentiation. Nature (2008) 455:1129–33. 10.1038/nature07443 PMC4051433 18948956

[64] Stecca B Ruiz i Altaba A A GLI1-p53 inhibitory loop controls neural stem cell and tumour cell numbers. EMBO J (2009) 28:663–76. 10.1038/emboj.2009.16 PMC2647769 19214186

[65] Po A Ferretti E Miele E De Smaele E Paganelli A Canettieri G Hedgehog controls neural stem cells through p53-independent regulation of Nanog. EMBO J (2010) 29:2646–58. 10.1038/emboj.2010.131 PMC2928686 20581804

[66] Zbinden M Duquet A Lorente-Trigos A Ngwabyt S-N Borges I Ruiz i Altaba A NANOG regulates glioma stem cells and is essential in vivo acting in a cross-functional network with GLI1 and p53. EMBO J (2010) 29:2659–74. 10.1038/emboj.2010.137 PMC2928692 20581802

[67] Moon JH Kwon S Jun EK Kim A Whang KY Kim H Nanog-induced dedifferentiation of p53-deficient mouse astrocytes into brain cancer stem-like cells. Biochem Biophys Res Commun (2011) 412:175–81. 10.1016/j.bbrc.2011.07.070 21810410

[68] Armesilla-Diaz A Bragado P Del Valle I Cuevas E Lazaro I Martin C p53 regulates the self-renewal and differentiation of neural precursors. Neuroscience (2009) 158:1378–89. 10.1016/j.neuroscience.2008.10.052 19038313

[69] Molchadsky A Shats I Goldfinger N Pevsner-Fischer M Olson M Rinon A p53 plays a role in mesenchymal differentiation programs, in a cell fate dependent manner. PloS One (2008) 3:e3707–7. 10.1371/journal.pone.0003707 PMC2577894 19002260

[70] Armesilla-Diaz A Elvira G Silva A p53 regulates the proliferation, differentiation and spontaneous transformation of mesenchymal stem cells. Exp Cell Res (2009) 315:3598–610. 10.1016/j.yexcr.2009.08.004 19686735

[71] He Y de Castro LF Shin MH Dubois W Yang HH Jiang S p53 loss increases the osteogenic differentiation of bone marrow stromal cells. Stem Cells (2015) 33:1304–19. 10.1002/stem.1925 PMC4376591 25524638

[72] Park EK Lee JC Park JW Bang SY Yi SA Kim BK Transcriptional repression of cancer stem cell marker CD133 by tumor suppressor p53. Cell Death Dis (2015) 6:e1964. 10.1038/cddis.2015.313 26539911 PMC4670923

[73] Godar S Ince TA Bell GW Feldser D Donaher JL Bergh J Growth-inhibitory and tumor- suppressive functions of p53 depend on its repression of CD44 expression. Cell (2008) 134:62–73. 10.1016/j.cell.2008.06.006 18614011 PMC3222460

[74] Liu C Kelnar K Liu B Chen X Calhoun-Davis T Li H The microRNA miR-34a inhibits prostate cancer stem cells and metastasis by directly repressing CD44. Nat Med (2011) 17:211–5. 10.1038/nm.2284 PMC3076220 21240262

[75] Liu K Lee J Kim JY Wang L Tian Y Chan ST Mitophagy Controls the Activities of Tumor Suppressor p53 to Regulate Hepatic Cancer Stem Cells. Mol Cell (2017) 68:281–92.e5. 10.1016/j.molcel.2017.09.022 29033320 PMC5687282

[76] Flesken-Nikitin A Hwang CI Cheng CY Michurina TV Enikolopov G Nikitin AY Ovarian surface epithelium at the junction area contains a cancer-prone stem cell niche. Nature (2013) 495:241–5. 10.1038/nature11979 PMC3982379 23467088

[77] Colaluca IN Tosoni D Nuciforo P Senic-Matuglia F Galimberti V Viale G NUMB controls p53 tumour suppressor activity. Nature (2008) 451:76–80. 10.1038/nature06412 18172499

[78] Cicalese A Bonizzi G Pasi CE Faretta M Ronzoni S Giulini B The tumor suppressor p53 regulates polarity of self-renewing divisions in mammary stem cells. Cell (2009) 138:1083–95. 10.1016/j.cell.2009.06.048 19766563

[79] Arsic N Gadea G Lagerqvist EL Busson M Cahuzac N Brock C The p53 isoform Δ133p53β promotes cancer stem cell potential. Stem Cell Rep (2015) 4:531–40. 10.1016/j.stemcr.2015.02.001 PMC4400643 25754205

[80] Chin KV Ueda K Pastan I Gottesman MM Modulation of activity of the promoter of the human MDR1 gene by Ras and p53. Science (1992) 255:459–62. 10.1126/science.1346476 1346476

[81] Bunting KD ABC transporters as phenotypic markers and functional regulators of stem cells. Stem Cells (2002) 20:11–20. 10.1002/stem.200011 11796918

[82] Ji Q Hao X Zhang M Tang W Yang M Li L MicroRNA miR-34 Inhibits Human Pancreatic Cancer Tumor-Initiating Cells. PloS One (2009) 4:e6816. 10.1371/journal.pone.0006816 19714243 PMC2729376

[83] Aloni-Grinstein R Shetzer Y Kaufman T Rotter V p53: The barrier to cancer stem cell formation. FEBS Lett (2014) 588:2580–9. 10.1016/j.febslet.2014.02.011 24560790

[84] Mizuno H Spike BT Wahl GM Levine AJ Inactivation of p53 in breast cancers correlates with stem cell transcriptional signatures. Proc Natl Acad Sci USA (2010) 107:22745–50. 10.1073/pnas.1017001108 PMC3012457 21149740

[85] Miller LD Smeds J George J Vega VB Vergara L Ploner A An expression signature for p53 status in human breast cancer predicts mutation status, transcriptional effects, and patient survival. Proc Natl Acad Sci USA (2005) 102:13550–5. 10.1073/pnas.0506230102 PMC1197273 16141321

[86] de Cremoux P Salomon AV Liva S Dendale R Bouchind’homme B Martin E p53 mutation as a genetic trait of typical medullary breast carcinoma. J Natl Cancer Inst (1999) 91:641–3. 10.1093/jnci/91.7.641 10203285

[87] Kochhar R Howard EM Umbreit JN Lau SK Metaplastic breast carcinoma with squamous differentiation: molecular and clinical analysis of six cases. Breast J (2005) 11:367–9. 10.1111/j.1075-122X.2005.00031.x 16174166

[88] Pinho AV Rooman I Real FX p53-dependent regulation of growth, epithelial-mesenchymal transition and stemness in normal pancreatic epithelial cells. Cell Cycle (2011) 10:1312–21. 10.4161/cc.10.8.15363 21490434

[89] Singh SK Chen NM Hessmann E Siveke J Lahmann M Singh G Antithetical NFATc1-Sox2 and p53-miR200 signaling networks govern pancreatic cancer cell plasticity. EMBO J (2015) 34:517–30. 10.15252/embj.201489574 PMC4331005 25586376

[90] Zhao Z Zuber J Diaz-Flores E Lintault L Kogan SC Shannon K p53 loss promotes acute myeloid leukemia by enabling aberrant self-renewal. Genes Dev (2010) 24:1389–402. 10.1101/gad.1940710 PMC2895198 20595231

[91] Junttila MR Karnezis AN Garcia D Madriles F Kortlever RM Rostker F Selective activation of p53-mediated tumour suppression in high-grade tumours. Nature (2010) 468:567–71. 10.1038/nature09526 PMC3011233 21107427

[92] Tschaharganeh DF Xue W Calvisi DF Evert M Michurina TV Dow LE p53-Dependent Nestin Regulation Links Tumor Suppression to Cellular Plasticity in Liver Cancer. Cell (2016) 165:1546–7. 10.1016/j.cell.2016.05.058 PMC5119474 27259155

[93] Tao L Roberts AL Dunphy KA Bigelow C Yan H Jerry DJ Repression of mammary stem/progenitor cells by p53 is mediated by Notch and separable from apoptotic activity. Stem Cells (2011) 29:119–27. 10.1002/stem.552 PMC3404152 21280161

[94] Pietersen AM Evers B Prasad AA Tanger E Cornelissen-Steijger P Jonkers J Bmi1 Regulates Stem Cells and Proliferation and Differentiation of Committed Cells in Mammary Epithelium. Curr Biol (2008) 18:1094–9. 10.1016/j.cub.2008.06.070 18635350

[95] Rodriguez R Rubio R Masip M Catalina P Nieto A de la Cueva T Loss of p53 induces tumorigenesis in p21-deficient mesenchymal stem cells. Neoplasia (2009) 11:397–407. 10.1593/neo.81620 19308294 PMC2657886

[96] Mantovani F Collavin L Del Sal G Mutant p53 as a guardian of the cancer cell. Cell Death Differ (2019) 26:199–212. 10.1038/s41418-018-0246-9 30538286 PMC6329812

[97] Muller PAJ Vousden KH Mutant p53 in cancer: new functions and therapeutic opportunities. Cancer Cell (2014) 25:304–17. 10.1016/j.ccr.2014.01.021 PMC3970583 24651012

[98] Buttitta F Marchetti A Gadducci A Pellegrini S Morganti M Carnicelli V p53 alterations are predictive of chemoresistance and aggressiveness in ovarian carcinomas: a molecular and immunohistochemical study. Br J Cancer (1997) 75:230–5. 10.1038/bjc.1997.38 PMC2063269 9010031

[99] Lotem J Sachs L A mutant p53 antagonizes the deregulated c-myc-mediated enhancement of apoptosis and decrease in leukemogenicity. Proc Natl Acad Sci USA (1995) 92:9672–6. 10.1073/pnas.92.21.9672 PMC40864 7568195

[100] Scian MJ Stagliano KER Anderson MAE Hassan S Bowman M Miles MF Tumor-derived p53 mutants induce NF-kappaB2 gene expression. Mol Cell Biol (2005) 25:10097–110. 10.1128/MCB.25.22.10097-10110.2005 PMC1280285 16260623

[101] Wang X Chen J-X Liu J-P You C Liu Y-H Mao Q Gain of function of mutant TP53 in glioblastoma: prognosis and response to temozolomide. Ann Surg Oncol (2014) 21:1337–44. 10.1245/s10434-013-3380-0 24248532

[102] Kolukula VK Sahu G Wellstein A Rodriguez OC Preet A Iacobazzi V SLC25A1, or CIC, is a novel transcriptional target of mutant p53 and a negative tumor prognostic marker. Oncotarget (2014) 5:1212–25. 10.18632/oncotarget.1831 PMC4012738 24681808

[103] Krishnan SR Nair BC Sareddy GR Roy SS Natarajan M Suzuki T Novel role of PELP1 in regulating chemotherapy response in mutant p53-expressing triple negative breast cancer cells. Breast Cancer Res Treat (2015) 150:487–99. 10.1007/s10549-015-3339-x PMC4385448 25788226

[104] Alam SK Yadav VK Bajaj S Datta A Dutta SK Bhattacharyya M DNA damage-induced ephrin-B2 reverse signaling promotes chemoresistance and drives EMT in colorectal carcinoma harboring mutant p53. Cell Death Differ (2016) 23:707–22. 10.1038/cdd.2015.133 PMC4986638 26494468

[105] Kalo E Kogan-Sakin I Solomon H Bar-Nathan E Shay M Shetzer Y Mutant p53R273H attenuates the expression of phase 2 detoxifying enzymes and promotes the survival of cells with high levels of reactive oxygen species. J Cell Sci (2012) 125:5578–86. 10.1242/jcs.106815 22899716

[106] Li D Yallowitz A Ozog L Marchenko N A gain-of-function mutant p53-HSF1 feed forward circuit governs adaptation of cancer cells to proteotoxic stress. Cell Death Dis (2014) 5:e1194–4. 10.1038/cddis.2014.158 PMC4001312 24763051

[107] Gaiddon C Lokshin M Ahn J Zhang T Prives C A subset of tumor-derived mutant forms of p53 down-regulate p63 and p73 through a direct interaction with the p53 core domain. Mol Cell Biol (2001) 21:1874–87. 10.1128/MCB.21.5.1874-1887.2001 PMC86759 11238924

[108] Ali A Shah AS Ahmad A Gain-of-function of mutant p53: mutant p53 enhances cancer progression by inhibiting KLF17 expression in invasive breast carcinoma cells. Cancer Lett (2014) 354:87–96. 10.1016/j.canlet.2014.07.045 25111898

[109] Ali A Wang Z Fu J Ji L Liu J Li L Differential regulation of the REGγ-proteasome pathway by p53/TGF-β signalling and mutant p53 in cancer cells. Nat Commun (2013) 4:2667. 10.1038/ncomms3667 24157709 PMC3876931

[110] Huang X Zhang Y Tang Y Butler N Kim J Guessous F A novel PTEN/mutant p53/c-Myc/Bcl-XL axis mediates context-dependent oncogenic effects of PTEN with implications for cancer prognosis and therapy. Neoplasia (2013) 15:952–65. 10.1593/neo.13376 PMC3730046 23908595

[111] Donzelli S Fontemaggi G Fazi F Di Agostino S Padula F Biagioni F MicroRNA-128-2 targets the transcriptional repressor E2F5 enhancing mutant p53 gain of function. Cell Death Differ (2012) 19:1038–48. 10.1038/cdd.2011.190 PMC3354056 22193543

[112] Masciarelli S Fontemaggi G Di Agostino S Donzelli S Carcarino E Strano S Gain-of-function mutant p53 downregulates miR-223 contributing to chemoresistance of cultured tumor cells. Oncogene (2014) 33:1601–8. 10.1038/onc.2013.106 23584479

[113] Muller PAJ Caswell PT Doyle B Iwanicki MP Tan EH Karim S Mutant p53 Drives Invasion by Promoting Integrin Recycling. Cell (2009) 139:1327–41. 10.1016/j.cell.2009.11.026 20064378

[114] Adorno M Cordenonsi M Montagner M Dupont S Wong C Hann B A Mutant-p53/Smad Complex Opposes p63 to Empower TGFβ-Induced Metastasis. Cell (2009) 137:87–98. 10.1016/j.cell.2009.01.039 19345189

[115] Muller PAJ Trinidad AG Caswell PT Norman JC Vousden KH Mutant p53 regulates Dicer through p63-dependent and -independent mechanisms to promote an invasive phenotype. J Biol Chem (2014) 289:122–32. 10.1074/jbc.M113.502138 PMC3879536 24220032

[116] Martello G Rosato A Ferrari F Manfrin A Cordenonsi M Dupont S A MicroRNA targeting dicer for metastasis control. Cell (2010) 141:1195–207. 10.1016/j.cell.2010.05.017 20603000

[117] Subramanian M Francis P Bilke S Li XL Hara T Lu X A mutant p53/let-7i-axis-regulated gene network drives cell migration, invasion and metastasis. Oncogene (2015) 34:1094–104. 10.1038/onc.2014.46 PMC4391367 24662829

[118] Neilsen PM Noll JE Mattiske S Bracken CP Gregory PA Schulz RB Mutant p53 drives invasion in breast tumors through up-regulation of miR-155. Oncogene (2013) 32:2992–3000. 10.1038/onc.2012.305 22797073

[119] Tucci P Agostini M Grespi F Markert EK Terrinoni A Vousden KH Loss of p63 and its microRNA-205 target results in enhanced cell migration and metastasis in prostate cancer. Proc Natl Acad Sci (2012) 109:15312. 10.1073/pnas.1110977109 22949650 PMC3458363

[120] Dong P Karaayvaz M Jia N Kaneuchi M Hamada J Watari H Mutant p53 gain-of-function induces epithelial-mesenchymal transition through modulation of the miR-130b-ZEB1 axis. Oncogene (2013) 32:3286–95. 10.1038/onc.2012.334 PMC3705163 22847613

[121] Wang W Cheng B Miao L Mei Y Wu M Mutant p53-R273H gains new function in sustained activation of EGFR signaling via suppressing miR-27a expression. Cell Death Dis (2013) 4:e574–4. 10.1038/cddis.2013.97 PMC3642368 23559009

[122] Weissmueller S Manchado E Saborowski M Morris JPT Wagenblast E Davis CA Mutant p53 drives pancreatic cancer metastasis through cell-autonomous PDGF receptor β signaling. Cell (2014) 157:382–94. 10.1016/j.cell.2014.01.066 PMC4001090 24725405

[123] Xiong S Tu H Kollareddy M Pant V Li Q Zhang Y Pla2g16 phospholipase mediates gain-of-function activities of mutant p53. Proc Natl Acad Sci USA (2014) 111:11145–50. 10.1073/pnas.1404139111 PMC4121829 25024203

[124] Kollareddy M Dimitrova E Vallabhaneni KC Chan A Le T Chauhan KM Regulation of nucleotide metabolism by mutant p53 contributes to its gain-of-function activities. Nat Commun (2015) 6:7389–9. 10.1038/ncomms8389 PMC4467467 26067754

[125] Arjonen A Kaukonen R Mattila E Rouhi P Högnäs G Sihto H Mutant p53-associated myosin-X upregulation promotes breast cancer invasion and metastasis. J Clin Invest (2014) 124:1069–82. 10.1172/JCI67280 PMC3934176 24487586

[126] Freed-Pastor WA Mizuno H Zhao X Langerød A Moon S-H Rodriguez-Barrueco R Mutant p53 Disrupts Mammary Tissue Architecture via the Mevalonate Pathway. Cell (2012) 148:244–58. 10.1016/j.cell.2011.12.017 PMC3511889 22265415

[127] Capaci V Bascetta L Fantuz M Beznoussenko GV Sommaggio R Cancila V Mutant p53 induces Golgi tubulo-vesiculation driving a prometastatic secretome. Nat Commun (2020) 11:3945. 10.1038/s41467-020-17596-5 32770028 PMC7414119

[128] Khromova NV Kopnin PB Stepanova EV Agapova LS Kopnin BP p53 hot-spot mutants increase tumor vascularization via ROS-mediated activation of the HIF1/VEGF-A pathway. Cancer Lett (2009) 276:143–51. 10.1016/j.canlet.2008.10.049 19091459

[129] Fontemaggi G Dell’Orso S Trisciuoglio D Shay T Melucci E Fazi F The execution of the transcriptional axis mutant p53, E2F1 and ID4 promotes tumor neo-angiogenesis. Nat Struct Mol Biol (2009) 16:1086–93. 10.1038/nsmb.1669 19783986

[130] Zhou G Wang J Zhao M Xie T-X Tanaka N Sano D Gain-of-function mutant p53 promotes cell growth and cancer cell metabolism via inhibition of AMPK activation. Mol Cell (2014) 54:960–74. 10.1016/j.molcel.2014.04.024 PMC4067806 24857548

[131] Zhang C Liu J Liang Y Wu R Zhao Y Hong X Tumour-associated mutant p53 drives the Warburg effect. Nat Commun (2013) 4:2935–5. 10.1038/ncomms3935 PMC3969270 24343302

[132] Freed-Pastor WA Mizuno H Zhao X Langerød A Moon S-H Rodriguez-Barrueco R Mutant p53 disrupts mammary tissue architecture via the mevalonate pathway. Cell (2012) 148:244–58. 10.1016/j.cell.2011.12.017 PMC3511889 22265415

[133] Frazier MW He X Wang J Gu Z Cleveland JL Zambetti GP Activation of c-myc gene expression by tumor-derived p53 mutants requires a discrete C-terminal domain. Mol Cell Biol (1998) 18:3735–43. 10.1128/MCB.18.7.3735 PMC108956 9632756

[134] Deb S Jackson CT Subler MA Martin DW Modulation of cellular and viral promoters by mutant human p53 proteins found in tumor cells. J Virol (1992) 66:6164–70. 10.1128/JVI.66.10.6164-6170.1992 PMC283665 1356162

[135] Ludes-Meyers JH Subler MA Shivakumar CV Munoz RM Jiang P Bigger JE Transcriptional activation of the human epidermal growth factor receptor promoter by human p53. Mol Cell Biol (1996) 16:6009–19. 10.1128/MCB.16.11.6009 PMC231603 8887630

[136] Vaughan CA Singh S Windle B Yeudall WA Frum R Grossman SR Gain-of-Function Activity of Mutant p53 in Lung Cancer through Up-Regulation of Receptor Protein Tyrosine Kinase Axl. Genes Cancer (2012) 3:491–502. 10.1177/1947601912462719 23264849 PMC3527987

[137] Di Como CJ Gaiddon C Prives C p73 Function Is Inhibited by Tumor-Derived p53 Mutants in Mammalian Cells. Mol Cell Biol (1999) 19:1438. 10.1128/MCB.19.2.1438 9891077 PMC116072

[138] Irwin MS Kondo K Marin MC Cheng LS Hahn WC Kaelin WG Jr. Chemosensitivity linked to p73 function. Cancer Cell (2003) 3:403–10. 10.1016/S1535-6108(03)00078-3 12726865

[139] Alexandrova EM Yallowitz AR Li D Xu S Schulz R Proia DA Improving survival by exploiting tumour dependence on stabilized mutant p53 for treatment. Nature (2015) 523:352–6. 10.1038/nature14430 PMC4506213 26009011

[140] Valenzuela MS Hu L Lueders J Walker R Meltzer PS Broader utilization of origins of DNA replication in cancer cell lines along a 78 kb region of human chromosome 2q34. J Cell Biochem (2012) 113:132–40. 10.1002/jcb.23336 PMC3590909 21898540

[141] Polotskaia A Xiao G Reynoso K Martin C Qiu W-G Hendrickson RC Proteome-wide analysis of mutant p53 targets in breast cancer identifies new levels of gain-of-function that influence PARP, PCNA, and MCM4. Proc Natl Acad Sci USA (2015) 112:E1220–9. 10.1073/pnas.1416318112 PMC4371979 25733866

[142] Xiao G Lundine D Annor GK Canar J Ellison V Polotskaia A Gain-of-Function Mutant p53 R273H Interacts with Replicating DNA and PARP1 in Breast Cancer. Cancer Res (2020) 80:394. 10.1158/0008-5472.CAN-19-1036 31776133 PMC7002183

[143] Datta A Ghatak D Das S Banerjee T Paul A Butti R p53 gain-of-function mutations increase Cdc7-dependent replication initiation. EMBO Rep (2017) 18:2030–50. 10.15252/embr.201643347 PMC5666604 28887320

[144] Liu K Lin F-T Graves JD Lee Y-J Lin W-C Mutant p53 perturbs DNA replication checkpoint control through TopBP1 and Treslin. Proc Natl Acad Sci (2017) 114:E3766. 10.1073/pnas.1619832114 28439015 PMC5441733

[145] Roy S Tomaszowski K-H Luzwick JW Park S Li J Murphy M p53 orchestrates DNA replication restart homeostasis by suppressing mutagenic RAD52 and POLθ pathways. eLife (2018) 7:e31723. 10.7554/eLife.31723 29334356 PMC5832412

[146] Samassekou O Bastien N Lichtensztejn D Yan J Mai S Drouin R Different TP53 mutations are associated with specific chromosomal rearrangements, telomere length changes, and remodeling of the nuclear architecture of telomeres. Genes Chromosomes Cancer (2014) 53:934–50. 10.1002/gcc.22205 25059482

[147] Hanel W Moll UM Links between mutant p53 and genomic instability. J Cell Biochem (2012) 113:433–9. 10.1002/jcb.23400 PMC4407809 22006292

[148] Bajaj S Alam SK Roy KS Datta A Nath S Roychoudhury S E2 Ubiquitin-conjugating Enzyme, UBE2C Gene, Is Reciprocally Regulated by Wild-type and Gain-of-Function Mutant p53. J Biol Chem (2016) 291:14231–47. 10.1074/jbc.M116.731398 PMC4933179 27129209

[149] Valenti F Ganci F Fontemaggi G Sacconi A Strano S Blandino G Gain of function mutant p53 proteins cooperate with E2F4 to transcriptionally downregulate RAD17 and BRCA1 gene expression. Oncotarget (2015) 6:5547–66. 10.18632/oncotarget.2587 PMC4467386 25650659

[150] Scian MJ Stagliano KER Deb D Ellis MA Carchman EH Das A Tumor-derived p53 mutants induce oncogenesis by transactivating growth-promoting genes. Oncogene (2004) 23:4430–43. 10.1038/sj.onc.1207553 15077194

[151] Hanahan D Weinberg RA Hallmarks of Cancer: The Next Generation. Cell (2011) 144:646–74. 10.1016/j.cell.2011.02.013 21376230

[152] Gudkov AV Gurova KV Komarova EA Inflammation and p53: A Tale of Two Stresses. Genes Cancer (2011) 2:503–16. 10.1177/1947601911409747 PMC3135644 21779518

[153] Weisz L Damalas A Liontos M Karakaidos P Fontemaggi G Maor-Aloni R Mutant p53 enhances nuclear factor kappaB activation by tumor necrosis factor alpha in cancer cells. Cancer Res (2007) 67:2396–401. 10.1158/0008-5472.CAN-06-2425 17363555

[154] Cooks T Pateras IS Tarcic O Solomon H Schetter AJ Wilder S Mutant p53 prolongs NF-κB activation and promotes chronic inflammation and inflammation-associated colorectal cancer. Cancer Cell (2013) 23:634–46. 10.1016/j.ccr.2013.03.022 PMC3657134 23680148

[155] Ubertini V Norelli G D’Arcangelo D Gurtner A Cesareo E Baldari S Mutant p53 gains new function in promoting inflammatory signals by repression of the secreted interleukin-1 receptor antagonist. Oncogene (2015) 34:2493–504. 10.1038/onc.2014.191 24998848

[156] Escobar-Hoyos LF Penson A Kannan R Cho H Pan C-H Singh RK Altered RNA Splicing by Mutant p53 Activates Oncogenic RAS Signaling in Pancreatic Cancer. Cancer Cell (2020) 38:198–211.e8. 10.1016/j.ccell.2020.05.010 32559497 PMC8028848

[157] Lang GA Iwakuma T Suh YA Liu G Rao VA Parant JM Gain of function of a p53 hot spot mutation in a mouse model of Li-Fraumeni syndrome. Cell (2004) 119:861–72. 10.1016/j.cell.2004.11.006 15607981

[158] Terzian T Suh YA Iwakuma T Post SM Neumann M Lang GA The inherent instability of mutant p53 is alleviated by Mdm2 or p16INK4a loss. Genes Dev (2008) 22:1337–44. 10.1101/gad.1662908 PMC2377188 18483220

[159] Suh Y-A Post SM Elizondo-Fraire AC Maccio DR Jackson JG El-Naggar AK Multiple stress signals activate mutant p53 in vivo. Cancer Res (2011) 71:7168–75. 10.1158/0008-5472.CAN-11-0459 PMC3320147 21983037

[160] Fiorini C Cordani M Padroni C Blandino G Di Agostino S Donadelli M Mutant p53 stimulates chemoresistance of pancreatic adenocarcinoma cells to gemcitabine. Biochim Biophys Acta (2015) 1853:89–100. 10.1016/j.bbamcr.2014.10.003 25311384

[161] Song H Hollstein M Xu Y p53 gain-of-function cancer mutants induce genetic instability by inactivating ATM. Nat Cell Biol (2007) 9:573–80. 10.1038/ncb1571 17417627

[162] Melnikova VO Santamaria AB Bolshakov SV Ananthaswamy HN Mutant p53 is constitutively phosphorylated at Serine 15 in UV-induced mouse skin tumors: involvement of ERK1/2 MAP kinase. Oncogene (2003) 22:5958–66. 10.1038/sj.onc.1206595 12955074

[163] Zerbini LF Wang Y Correa RG Cho JY Libermann TA Blockage of NF-kappaB induces serine 15 phosphorylation of mutant p53 by JNK kinase in prostate cancer cells. Cell Cycle (2005) 4:1247–53. 10.4161/cc.4.9.1966 16082226

[164] Sonego M Schiappacassi M Lovisa S Dall’Acqua A Bagnoli M Lovat F Stathmin regulates mutant p53 stability and transcriptional activity in ovarian cancer. EMBO Mol Med (2014) 6:295–5. 10.1002/emmm.201470020 PMC3662314 23610071

[165] Valenti F Fausti F Biagioni F Shay T Fontemaggi G Domany E Mutant p53 oncogenic functions are sustained by Plk2 kinase through an autoregulatory feedback loop. Cell Cycle (2011) 10:4330–40. 10.4161/cc.10.24.18682 22134238

[166] Bode AM Dong Z Post-translational modification of p53 in tumorigenesis. Nat Rev Cancer (2004) 4:793–805. 10.1038/nrc1455 15510160

[167] Minamoto T Buschmann T Habelhah H Matusevich E Tahara H Boerresen-Dale A-L Distinct pattern of p53 phosphorylation in human tumors. Oncogene (2001) 20:3341–7. 10.1038/sj.onc.1204458 11423984

[168] Warnock LJ Raines SA Milner J Aurora A mediates cross-talk between N- and C-terminal post-translational modifications of p53. Cancer Biol Ther (2011) 12:1059–68. 10.4161/cbt.12.12.18141 PMC3335940 22157150

[169] Jethwa A Słabicki M Hüllein J Jentzsch M Dalal V Rabe S TRRAP is essential for regulating the accumulation of mutant and wild-type p53 in lymphoma. Blood (2018) 131:2789–802. 10.1182/blood-2017-09-806679 29653964

[170] Murr R Vaissière T Sawan C Shukla V Herceg Z Orchestration of chromatin-based processes: mind the TRRAP. Oncogene (2007) 26:5358–72. 10.1038/sj.onc.1210605 17694078

[171] Knowell AE Patel D Morton DJ Sharma P Glymph S Chaudhary J Id4 dependent acetylation restores mutant-p53 transcriptional activity. Mol Cancer (2013) 12:161. 10.1186/1476-4598-12-161 24330748 PMC3866570

[172] Rodriguez OC Choudhury S Kolukula V Vietsch EE Catania J Preet A Dietary downregulation of mutant p53 levels via glucose restriction: mechanisms and implications for tumor therapy. Cell Cycle (2012) 11:4436–46. 10.4161/cc.22778 PMC3552926 23151455

[173] Yi YW Kang HJ Kim HJ Kong Y Brown ML Bae I Targeting mutant p53 by a SIRT1 activator YK-3-237 inhibits the proliferation of triple-negative breast cancer cells. Oncotarget (2013) 4:984–94. 10.18632/oncotarget.1070 PMC3759676 23846322

[174] Li M Brooks CL Wu-Baer F Chen D Baer R Gu W Mono- Versus Polyubiquitination: Differential Control of p53 Fate by Mdm2. Science (2003) 302:1972. 10.1126/science.1091362 14671306

[175] Hainaut P Milner J Interaction of heat-shock protein 70 with p53 translated in vitro: evidence for interaction with dimeric p53 and for a role in the regulation of p53 conformation. EMBO J (1992) 11:3513–20. 10.1002/j.1460-2075.1992.tb05434.x PMC556809 1396554

[176] Sugito K Yamane M Hattori H Hayashi Y Tohnai I Ueda M Interaction between hsp70 and hsp40, eukaryotic homologues of DnaK and DnaJ, in human cells expressing mutant-type p53. FEBS Lett (1995) 358:161–4. 10.1016/0014-5793(94)01417-Y 7828728

[177] Blagosklonny MV Toretsky J Bohen S Neckers L Mutant conformation of p53 translated in vitro or in vivo requires functional HSP90. Proc Natl Acad Sci USA (1996) 93:8379–83. 10.1073/pnas.93.16.8379 PMC38679 8710879

[178] Peng Y Chen L Li C Lu W Chen J Inhibition of MDM2 by hsp90 contributes to mutant p53 stabilization. J Biol Chem (2001) 276:40583–90. 10.1074/jbc.M102817200 11507088

[179] Li D Marchenko ND Schulz R Fischer V Velasco-Hernandez T Talos F Functional inactivation of endogenous MDM2 and CHIP by HSP90 causes aberrant stabilization of mutant p53 in human cancer cells. Mol Cancer Res (2011) 9:577–88. 10.1158/1541-7786.MCR-10-0534 PMC3097033 21478269

[180] Ingallina E Sorrentino G Bertolio R Lisek K Zannini A Azzolin L Mechanical cues control mutant p53 stability through a mevalonate–RhoA axis. Nat Cell Biol (2018) 20:28–35. 10.1038/s41556-017-0009-8 29255172 PMC6179142

[181] Muller P Hrstka R Coomber D Lane DP Vojtesek B Chaperone-dependent stabilization and degradation of p53 mutants. Oncogene (2008) 27:3371–83. 10.1038/sj.onc.1211010 18223694

[182] Finlay CA Hinds PW Tan TH Eliyahu D Oren M Levine AJ Activating mutations for transformation by p53 produce a gene product that forms an hsc70-p53 complex with an altered half-life. Mol Cell Biol (1988) 8:531–9. 10.1128/MCB.8.2.531 PMC363177 2832726

[183] Lu WJ Lee NP Kaul SC Lan F Poon RTP Wadhwa R Mortalin–p53 interaction in cancer cells is stress dependent and constitutes a selective target for cancer therapy. Cell Death Differ (2011) 18:1046–56. 10.1038/cdd.2010.177 PMC3131943 21233847

[184] Yue X Zhao Y Liu J Zhang C Yu H Wang J BAG2 promotes tumorigenesis through enhancing mutant p53 protein levels and function. Elife (2015) 4:1–23. 10.7554/eLife.08401 PMC4561369 26271008

[185] Yue X Zhao Y Huang G Li J Zhu J Feng Z A novel mutant p53 binding partner BAG5 stabilizes mutant p53 and promotes mutant p53 GOFs in tumorigenesis. Cell Discovery (2016) 2:16039. 10.1038/celldisc.2016.39 27807478 PMC5088412

[186] Sarig R Rivlin N Brosh R Bornstein C Kamer I Ezra O Mutant p53 facilitates somatic cell reprogramming and augments the malignant potential of reprogrammed cells. J Exp Med (2010) 207:2127–40. 10.1084/jem.20100797 PMC2947075 20696700

[187] Grespi F Landré V Molchadsky A Di Daniele N Marsella LT Melino G Differential regulated microRNA by wild type and mutant p53 in induced pluripotent stem cells. Cell Death Dis (2016) 7:e2567–7. 10.1038/cddis.2016.419 PMC5260988 28032868

[188] Sorrentino G Ruggeri N Specchia V Cordenonsi M Mano M Dupont S Metabolic control of YAP and TAZ by the mevalonate pathway. Nat Cell Biol (2014) 16:357–66. 10.1038/ncb2936 24658687

[189] Datta A Das P Dey S Ghuwalewala S Ghatak D Alam SK Genome-Wide Small RNA Sequencing Identifies MicroRNAs Deregulated in Non-Small Cell Lung Carcinoma Harboring Gain-of-Function Mutant p53. Genes (2019) 10:852. 10.3390/genes10110852 PMC6895929 31661871

[190] Nieto MA Epithelial plasticity: a common theme in embryonic and cancer cells. Science (2013) 342:1234850. 10.1126/science.1234850 24202173

[191] Thiery JP Acloque H Huang RY Nieto MA Epithelial-mesenchymal transitions in development and disease. Cell (2009) 139:871–90. 10.1016/j.cell.2009.11.007 19945376

[192] Peinado H Olmeda D Cano A Snail, Zeb and bHLH factors in tumour progression: an alliance against the epithelial phenotype? Nat Rev Cancer (2007) 7:415–28. 10.1038/nrc2131 17508028

[193] Ocaña OH Córcoles R Fabra A Moreno-Bueno G Acloque H Vega S Metastatic colonization requires the repression of the epithelial-mesenchymal transition inducer Prrx1. Cancer Cell (2012) 22:709–24. 10.1016/j.ccr.2012.10.012 23201163

[194] Chaffer CL Weinberg RA A Perspective on Cancer Cell Metastasis. Science (2011) 331:1559. 10.1126/science.1203543 21436443

[195] Brabletz T Jung A Spaderna S Hlubek F Kirchner T Migrating cancer stem cells — an integrated concept of malignant tumour progression. Nat Rev Cancer (2005) 5:744–9. 10.1038/nrc1694 16148886

[196] Mani SA Guo W Liao M-J Eaton EN Ayyanan A Zhou AY The epithelial-mesenchymal transition generates cells with properties of stem cells. Cell (2008) 133:704–15. 10.1016/j.cell.2008.03.027 PMC2728032 18485877

[197] Morel A-P Lièvre M Thomas C Hinkal G Ansieau S Puisieux A Generation of breast cancer stem cells through epithelial-mesenchymal transition. PLoS One (2008) 3:e2888–8. 10.1371/journal.pone.0002888 PMC2492808 18682804

[198] Chang CJ Chao CH Xia W Yang JY Xiong Y Li CW p53 regulates epithelial-mesenchymal transition and stem cell properties through modulating miRNAs. Nat Cell Biol (2011) 13:317–23. 10.1038/ncb2173 PMC3075845 21336307

[199] Burk U Schubert J Wellner U Schmalhofer O Vincan E Spaderna S A reciprocal repression between ZEB1 and members of the miR-200 family promotes EMT and invasion in cancer cells. EMBO Rep (2008) 9:582–9. 10.1038/embor.2008.74 PMC2396950 18483486

[200] Park SM Gaur AB Lengyel E Peter ME The miR-200 family determines the epithelial phenotype of cancer cells by targeting the E-cadherin repressors ZEB1 and ZEB2. Genes Dev (2008) 22:894–907. 10.1101/gad.1640608 18381893 PMC2279201

[201] Shimono Y Zabala M Cho RW Lobo N Dalerba P Qian D Downregulation of miRNA-200c Links Breast Cancer Stem Cells with Normal Stem Cells. Cell (2009) 138:592–603. 10.1016/j.cell.2009.07.011 19665978 PMC2731699

[202] Ren D Wang M Guo W Zhao X Tu XA Huang S Wild-type p53 suppresses the epithelial-mesenchymal transition and stemness in PC-3 prostate cancer cells by modulating miR−145. Int J Oncol (2013) 42:1473–81. 10.3892/ijo.2013.1825 23404342

[203] Zhao Y Li Y Sheng J Wu F Li K Huang R P53-R273H mutation enhances colorectal cancer stemness through regulating specific lncRNAs. J Exp Clin Cancer Res (2019) 38:379. 10.1186/s13046-019-1375-9 31455383 PMC6712617

[204] Begicevic R-R Falasca M ABC Transporters in Cancer Stem Cells: Beyond Chemoresistance. Int J Mol Sci (2017) 18:2362. 10.3390/ijms18112362 PMC5713331 29117122

[205] Zhou X Hao Q Lu H Mutant p53 in cancer therapy-the barrier or the path. J Mol Cell Biol (2019) 11:293–305. 10.1093/jmcb/mjy072 30508182 PMC6487791

[206] Wang Y-H Scadden DT Harnessing the apoptotic programs in cancer stem-like cells. EMBO Rep (2015) 16:1084–98. 10.15252/embr.201439675 PMC4576979 26253117

[207] Schulz A Meyer F Dubrovska A Borgmann K Cancer Stem Cells and Radioresistance: DNA Repair and Beyond. Cancers (Basel) (2019) 11:862. 10.3390/cancers11060862 PMC6627210 31234336

[208] Shetzer Y Kagan S Koifman G Sarig R Kogan-Sakin I Charni M The onset of p53 loss of heterozygosity is differentially induced in various stem cell types and may involve the loss of either allele. Cell Death Differ (2014) 21:1419–31. 10.1038/cdd.2014.57 PMC4131174 24832469

[209] Weisz L Damalas A Liontos M Karakaidos P Fontemaggi G Maor-Aloni R Mutant p53 Enhances Nuclear Factor κB Activation by Tumor Necrosis Factor α in Cancer Cells. Cancer Res (2007) 67:2396–401. 10.1158/0008-5472.CAN-06-2425 17363555

[210] Shigdar S Li Y Bhattacharya S O’Connor M Pu C Lin J Inflammation and cancer stem cells. Cancer Lett (2014) 345:271–8. 10.1016/j.canlet.2013.07.031 23941828

[211] Zhao Y Bao Q Renner A Camaj P Eichhorn M Ischenko I Cancer stem cells and angiogenesis. Int J Dev Biol (2011) 55:477–82. 10.1387/ijdb.103225yz 21732274

[212] Bao S Wu Q Sathornsumetee S Hao Y Li Z Hjelmeland AB Stem Cell–like Glioma Cells Promote Tumor Angiogenesis through Vascular Endothelial Growth Factor. Cancer Res (2006) 66:7843–8. 10.1158/0008-5472.CAN-06-1010 16912155

[213] Shipitsin M Polyak K The cancer stem cell hypothesis: in search of definitions, markers, and relevance. Lab Invest (2008) 88:459–63. 10.1038/labinvest.2008.14 PMC3702270 18379567

[214] Clevers H The cancer stem cell: premises, promises and challenges. Nat Med (2011) 17:313–9. 10.1038/nm.2304 21386835

[215] Zhao Y Dong Q Li J Zhang K Qin J Zhao J Targeting cancer stem cells and their niche: perspectives for future therapeutic targets and strategies. Semin Cancer Biol (2018) 53:139–55. 10.1016/j.semcancer.2018.08.002 30081228

[216] Chen W Dong J Haiech J Kilhoffer M-C Zeniou M Cancer Stem Cell Quiescence and Plasticity as Major Challenges in Cancer Therapy. Stem Cells Int (2016) 2016:1740936. 10.1155/2016/1740936 27418931 PMC4932171

[217] Zhao T Xu Y p53 and stem cells: new developments and new concerns. Trends Cell Biol (2010) 20:170–5. 10.1016/j.tcb.2009.12.004 20061153

[218] Krizhanovsky V Lowe SW The promises and perils of p53. Nature (2009) 460:1085–6. 10.1038/4601085a PMC2974062 19713919

[219] Blandino G Di Agostino S New therapeutic strategies to treat human cancers expressing mutant p53 proteins. J Exp Clin Cancer Res (2018) 37 30:1–13. 10.1186/s13046-018-0705-7 29448954 PMC5815234

[220] Prabhu VV Allen JE Hong B Zhang S Cheng H El-Deiry WS Therapeutic targeting of the p53 pathway in cancer stem cells. Expert Opin Ther Targets (2012) 16:1161–74. 10.1517/14728222.2012.726985 PMC3650610 22998602

[221] Bykov VJN Eriksson SE Bianchi J Wiman KG Targeting mutant p53 for efficient cancer therapy. Nat Rev Cancer (2018) 18:89–102. 10.1038/nrc.2017.109 29242642

[222] Buhaescu I Izzedine H Mevalonate pathway: a review of clinical and therapeutical implications. Clin Biochem (2007) 40:575–84. 10.1016/j.clinbiochem.2007.03.016 17467679

[223] Panciera T Azzolin L Cordenonsi M Piccolo S Mechanobiology of YAP and TAZ in physiology and disease. Nat Rev Mol Cell Biol (2017) 18:758–70. 10.1038/nrm.2017.87 PMC6192510 28951564

[224] Li Z Wang Y Zhu Y Yuan C Wang D Zhang W The Hippo transducer TAZ promotes epithelial to mesenchymal transition and cancer stem cell maintenance in oral cancer. Mol Oncol (2015) 9:1091–105. 10.1016/j.molonc.2015.01.007 PMC5528756 25704916

[225] Zanconato F Cordenonsi M Piccolo S YAP/TAZ at the Roots of Cancer. Cancer Cell (2016) 29:783–803. 10.1016/j.ccell.2016.05.005 27300434 PMC6186419

[226] Di Agostino S Sorrentino G Ingallina E Valenti F Ferraiuolo M Bicciato S YAP enhances the pro-proliferative transcriptional activity of mutant p53 proteins. EMBO Rep (2016) 17:188–201. 10.15252/embr.201540488 26691213 PMC5290815

[227] Cordani M Oppici E Dando I Butturini E Dalla Pozza E Nadal-Serrano M Mutant p53 proteins counteract autophagic mechanism sensitizing cancer cells to mTOR inhibition. Mol Oncol (2016) 10:1008–29. 10.1016/j.molonc.2016.04.001 PMC5423176 27118659

[228] Yadav UP Singh T Kumar P Sharma P Kaur H Sharma S Metabolic Adaptations in Cancer Stem Cells. Front Oncol (2020) 10:1–19 10.3389/fonc.2020.01010 32670883 PMC7330710

[229] Mantovani F Walerych D Sal GD Targeting mutant p53 in cancer: a long road to precision therapy. FEBS J (2017) 284:837–50. 10.1111/febs.13948 27808469

[230] Pollak MN Investigating metformin for cancer prevention and treatment: the end of the beginning. Cancer Discovery (2012) 2:778–90. 10.1158/2159-8290.CD-12-0263 22926251

[231] Rustighi A Zannini A Tiberi L Sommaggio R Piazza S Sorrentino G Prolyl-isomerase Pin1 controls normal and cancer stem cells of the breast. EMBO Mol Med (2014) 6:99–119. 10.1002/emmm.201302909 24357640 PMC3936488

[232] Girardini JE Napoli M Piazza S Rustighi A Marotta C Radaelli E A Pin1/mutant p53 axis promotes aggressiveness in breast cancer. Cancer Cell (2011) 20:79–91. 10.1016/j.ccr.2011.06.004 21741598

[233] Wei S Kozono S Kats L Nechama M Li W Guarnerio J Active Pin1 is a key target of all-trans retinoic acid in acute promyelocytic leukemia and breast cancer. Nat Med (2015) 21:457–66. 10.1038/nm.3839 PMC4425616 25849135

[234] Bouchie A First microRNA mimic enters clinic. Nat Biotechnol (2013) 31:577. 10.1038/nbt0713-577 23839128

[235] Ceppi P Peter ME MicroRNAs regulate both epithelial-to-mesenchymal transition and cancer stem cells. Oncogene (2014) 33:269–78. 10.1038/onc.2013.55 23455327

[236] Zhang L Liao Y Tang L MicroRNA-34 family: a potential tumor suppressor and therapeutic candidate in cancer. J Exp Clin Cancer Res (2019) 38:53. 10.1186/s13046-019-1059-5 30717802 PMC6360685

[237] Hong DS Kang Y-K Borad M Sachdev J Ejadi S Lim HY Phase 1 study of MRX34, a liposomal miR-34a mimic, in patients with advanced solid tumours. Br J Cancer (2020) 122:1630–7. 10.1038/s41416-020-0802-1 PMC7251107 32238921

[238] Deng J Yang M Jiang R An N Wang X Liu B Long Non-Coding RNA HOTAIR Regulates the Proliferation, Self-Renewal Capacity, Tumor Formation and Migration of the Cancer Stem-Like Cell (CSC) Subpopulation Enriched from Breast Cancer Cells. PLoS One (2017) 12:e0170860. 10.1371/journal.pone.0170860 28122024 PMC5266294

[239] Leijen S Beijnen JH Schellens JH Abrogation of the G2 checkpoint by inhibition of Wee-1 kinase results in sensitization of p53-deficient tumor cells to DNA-damaging agents. Curr Clin Pharmacol (2010) 5:186–91. 10.2174/157488410791498824 20406171

[240] De Witt Hamer PC Mir SE Noske D Van Noorden CJ Würdinger T WEE1 kinase targeting combined with DNA-damaging cancer therapy catalyzes mitotic catastrophe. Clin Cancer Res (2011) 17:4200–7. 10.1158/1078-0432.CCR-10-2537 21562035

[241] Sand A Piacsek M Donohoe DL Duffin AT Riddell GT Sun C WEE1 inhibitor, AZD1775, overcomes trastuzumab resistance by targeting cancer stem-like properties in HER2-positive breast cancer. Cancer Lett (2020) 472:119–31. 10.1016/j.canlet.2019.12.023 31866466

[242] Ferraiuolo M Di Agostino S Blandino G Strano S Oncogenic Intra-p53 Family Member Interactions in Human Cancers. Front Oncol (2016) 6:77. 10.3389/fonc.2016.00077 27066457 PMC4814729

[243] Vilgelm A Wei JX Piazuelo MB Washington MK Prassolov V El-Rifai W DeltaNp73alpha regulates MDR1 expression by inhibiting p53 function. Oncogene (2008) 27:2170–6. 10.1038/sj.onc.1210862 PMC4066670 17952118

[244] Zhang Y Yan W Jung YS Chen X Mammary epithelial cell polarity is regulated differentially by p73 isoforms via epithelial-to-mesenchymal transition. J Biol Chem (2012) 287:17746–53. 10.1074/jbc.M112.358143 PMC3366836 22457351

[245] Strano S Blandino G p73-Mediated Chemosensitivity: A Preferential Target of Oncogenic Mutant p53. Cell Cycle (2003) 2:345–6. 10.4161/cc.2.4.426 12851488

[246] Santini S Di Agostino S Coppari E Bizzarri AR Blandino G Cannistraro S Interaction of mutant p53 with p73: A Surface Plasmon Resonance and Atomic Force Spectroscopy study. Biochim Biophys Acta (BBA) Gen Subj (2014) 1840:1958–64. 10.1016/j.bbagen.2014.02.014 24576672

[247] Müller M Schleithoff ES Stremmel W Melino G Krammer PH Schilling T One, two, three–p53, p63, p73 and chemosensitivity. Drug Resist Update (2006) 9:288–306. 10.1016/j.drup.2007.01.001 17287142

[248] Li Y Prives C Are interactions with p63 and p73 involved in mutant p53 gain of oncogenic function? Oncogene (2007) 26:2220–5. 10.1038/sj.onc.1210311 17401431

[249] Muller PAJ Trinidad AG Timpson P Morton JP Zanivan S van den Berghe PVE Mutant p53 enhances MET trafficking and signalling to drive cell scattering and invasion. Oncogene (2013) 32:1252–65. 10.1038/onc.2012.148 PMC3592945 22580601

[250] Adorno M Cordenonsi M Montagner M Dupont S Wong C Hann B A Mutant-p53/Smad complex opposes p63 to empower TGFbeta-induced metastasis. Cell (2009) 137:87–98. 10.1016/j.cell.2009.01.039 19345189

[251] Kravchenko JE Ilyinskaya GV Komarov PG Agapova LS Kochetkov DV Strom E Small-molecule RETRA suppresses mutant p53-bearing cancer cells through a p73-dependent salvage pathway. Proc Natl Acad Sci (2008) 105:6302. 10.1073/pnas.0802091105 18424558 PMC2327210

[252] Lu C Wang W El-Deiry WS Non-genotoxic anti-neoplastic effects of ellipticine derivative NSC176327 in p53-deficient human colon carcinoma cells involve stimulation of p73. Cancer Biol Ther (2008) 7:2039–46. 10.4161/cbt.7.12.7461 PMC3626664 19106635

[253] Wang W Kim S-H El-Deiry WS Small-molecule modulators of p53 family signaling and antitumor effects in p53-deficient human colon tumor xenografts. Proc Natl Acad Sci USA (2006) 103:11003–8. 10.1073/pnas.0604507103 PMC1544164 16835297

[254] Rosenbluth JM Mays DJ Pino MF Tang LJ Pietenpol JA A gene signature-based approach identifies mTOR as a regulator of p73. Mol Cell Biol (2008) 28:5951–64. 10.1128/MCB.00305-08 PMC2547001 18678646

[255] Guida E Bisso A Fenollar-Ferrer C Napoli M Anselmi C Girardini JE Peptide Aptamers Targeting Mutant p53 Induce Apoptosis in Tumor Cells. Cancer Res (2008) 68:6550. 10.1158/0008-5472.CAN-08-0137 18701478

[256] Vijayakumaran R Tan KH Miranda PJ Haupt S Haupt Y Regulation of Mutant p53 Protein Expression. Front Oncol (2015) 5:284–4. 10.3389/fonc.2015.00284 PMC4681805 26734569

[257] Li D Marchenko ND Moll UM SAHA shows preferential cytotoxicity in mutant p53 cancer cells by destabilizing mutant p53 through inhibition of the HDAC6-Hsp90 chaperone axis. Cell Death Differ (2011) 18:1904–13. 10.1038/cdd.2011.71 PMC3170683 21637290

[258] Le HT Nguyen HT Min H-Y Hyun SY Kwon S Lee Y Panaxynol, a natural Hsp90 inhibitor, effectively targets both lung cancer stem and non-stem cells. Cancer Lett (2018) 412:297–307. 10.1016/j.canlet.2017.10.013 29061506

[259] Gandolfi S Laubach JP Hideshima T Chauhan D Anderson KC Richardson PG The proteasome and proteasome inhibitors in multiple myeloma. Cancer Metastasis Rev (2017) 36:561–84. 10.1007/s10555-017-9707-8 29196868

[260] Walerych D Lisek K Sommaggio R Piazza S Ciani Y Dalla E Proteasome machinery is instrumental in a common gain-of-function program of the p53 missense mutants in cancer. Nat Cell Biol (2016) 18:897–909. 10.1038/ncb3380 27347849

[261] Oshimori N Oristian D Fuchs E TGF-β promotes heterogeneity and drug resistance in squamous cell carcinoma. Cell (2015) 160:963–76. 10.1016/j.cell.2015.01.043 PMC4509607 25723170

[262] Buis J Wu Y Deng Y Leddon J Westfield G Eckersdorff M Mre11 nuclease activity has essential roles in DNA repair and genomic stability distinct from ATM activation. Cell (2008) 135:85–96. 10.1016/j.cell.2008.08.015 18854157 PMC2645868

[263] Restle A Färber M Baumann C Böhringer M Scheidtmann KH Müller-Tidow C Dissecting the role of p53 phosphorylation in homologous recombination provides new clues for gain-of-function mutants. Nucleic Acids Res (2008) 36:5362–75. 10.1093/nar/gkn503 PMC2532731 18697815

[264] Liu C Srihari S Cao K-AL Chenevix-Trench G Simpson PT Ragan MA A fine-scale dissection of the DNA double-strand break repair machinery and its implications for breast cancer therapy. Nucleic Acids Res (2014) 42:6106–27. 10.1093/nar/gku284 PMC4041457 24792170

[265] Jarrar A Lotti F DeVecchio J Ferrandon S Gantt G Mace A Poly(ADP-Ribose) Polymerase Inhibition Sensitizes Colorectal Cancer-Initiating Cells to Chemotherapy. Stem Cells (2019) 37:42–53. 10.1002/stem.2929 30353615

[266] Bellio C DiGloria C Foster R James K Konstantinopoulos PA Growdon WB PARP Inhibition Induces Enrichment of DNA Repair-Proficient CD133 and CD117 Positive Ovarian Cancer Stem Cells. Mol Cancer Res (2019) 17:431–45. 10.1158/1541-7786.MCR-18-0594 30401718

